# The (un)likelihood of clock-driven lateral root priming; a modeling exploration

**DOI:** 10.1093/plcell/koag213

**Published:** 2026-07-14

**Authors:** Kirsten H Ten Tusscher

**Affiliations:** Theoretical Biology, IBB, Department of Biology, Utrecht University, PO Box 80125, 3508 TC Utrecht, the Netherlands; Experimental and Computational Plant Development, IEB, Department of Biology, Utrecht University, PO Box 80125, 3508 TC Utrecht, the Netherlands

## Abstract

Lateral root formation starts with priming, predisposing subsets of pericycle cells with the potential of future lateral root formation through semi-periodic elevations in auxin (signaling). While various mechanisms have been suggested, one frequently suggested hypothesis proposes a genetic, cell-autonomous root clock akin to the clock underlying vertebrate somitogenesis. Still, although gene expression variations were observed, so far this clock has not been proven. From a functional and evolutionary perceptive, it is furthermore an open question whether lateral root priming is not more likely to arise from an emergent tissue level process similar to phyllotaxis. To help settle this debate in this study, I use general knowledge of oscillator dynamics and simple models of auxin signaling to underline the unlikelihood of a root clock driving lateral root priming. I show how, within a single cell, oscillations are limited to a small parameter domain, with constraints intensifying due to the presence of multiple AUX/IAA and ARF types as well as auxin export. Furthermore, I demonstrate how non-meristem–based oscillations, due to a lack of memory of oscillator phase, cannot drive periodic prebranch site formation, and periodic inputs are insufficient to induce phase differences. Combined, these factors underline the unlikelihood of a root clock driving lateral root priming.

## Introduction

**Figure 1:**
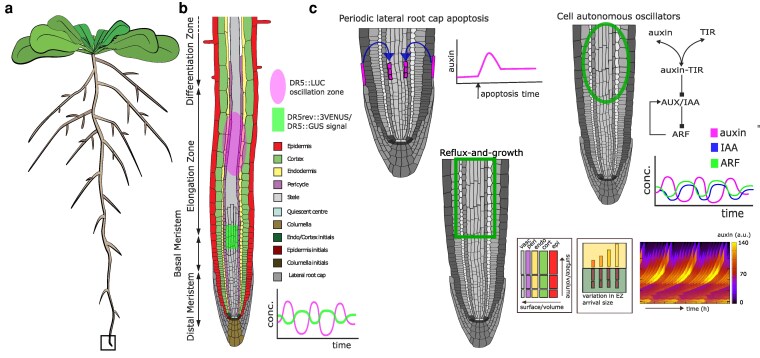
Various hypotheses for root system priming. a) A schematic of Arabidopsis thaliana rosette stage plant architecture, showing the branching of the root system through the formation of lateral roots. b) Zoom in of the boxed region in A the first signs of lateral root priming are observed just above the meristem while the oscillation zone appears to be localized more shootward. c) Three alternative hypothesis for the mechanism underlying lateral root priming, periodic root cap apoptosis, reflux-and-growth and a root clock.

Root system branching is a major determinant of a plant's access to water and nutrients as well as its capability to anchor to its substrate. Independent of whether a plant has a taproot or fibrous root system, either the single main root or the many equivalent fibrous roots further branch through the formation of lateral roots (Fig. 1a). Lateral root formation has been studied in most detail in the model plant *Arabidopsis thaliana*. Here the first steps in this process involve semi-periodic elevations in auxin (signaling) called priming that prepattern groups of cells to gain competence for future lateral root formation (De Smet et al. 2007; Moreno-Risueno et al. 2010) (Fig. 1b). Dependent on the level of auxin elevation, a so-called stable prebranch site is formed from which in later stages lateral root initiation, primordium formation and lateral root emergence may occur. For each of these steps in lateral root formation, as well as the subsequent rate and angle of outgrowth of the emerged root, auxin signaling and environmental conditions are major determinants (Lavenus et al. 2013; Santos Teixeira and Ten Tusscher 2019).

The elevations in auxin (signaling) involved in priming occur above the meristem, in the elongation zone, which has been dubbed the oscillation zone (Moreno-Risueno et al. 2010). More detailed measurements indicate that these elevations occur in the vasculature just above the meristem and become transduced to the neighboring xylem pole pericycle cells from which lateral root formation takes place higher up in the elongation zone (De Smet et al. 2007; De Gernier et al. 2025) (Fig. 1b). Experimental data also indicate that auxin transport and availability (De Smet et al. 2007; Xuan et al. 2015) play a major role in lateral root–priming amplitude, while the periodicity of the process strongly correlates with the process of shedding of uppermost lateral root cap cells (Xuan et al. 2016). Based on these findings, several models have been proposed that explain lateral root priming as a tissue-level property, without individual cells undergoing oscillations. In the case of periodic root cap apoptosis, the topmost dying root cap cell is hypothesized to extrude its auxin contents to the neighboring root tissues, with auxin subsequently being transported inwards to the vasculature (Xuan et al. 2016). In the reflected-flow model, feedback interactions between auxin and auxin transport rates in growing tissue give rise to periodic auxin peaks (Mironova et al. 2010). Finally, in the reflux-and-growth model, it is the root tip reflux loop that creates an auxin loading zone in the early meristem, while stem cell–driven growth results in periodic variations in cell sizes and thereby auxin loading capacity (van den Berg et al. 2021) (Fig. 1c). In the latter model, vasculature-localized priming naturally arises from the narrowness and hence high surface to volume ratio of vascular cells. In all 3 cases, priming arises as an emergent property of the interplay between tissue level auxin transport and growth. Notably, in phyllotaxis, auxin maxima have been shown to arise from the interplay between growth and auxin transport (Jönsson et al. 2006; Smith et al. 2006).

Other experimental data have shown that, concurrently with the elevations in auxin signaling, many genes show in- or out-of-phase changes in expression (Moreno-Risueno et al. 2010). Involved genes drive processes such as vesicle trafficking, cell wall pectin esterification (Wachsman et al. 2020), and retinal binding proteins (Dickinson et al. 2021) that affect cell length, oscillation amplitude, and lateral root formation success. The occurrence of periodic changes in gene expression led to the proposal of a so-called root clock, analogous to the well-established somitogenesis clock driving vertebrate axial patterning (Fig. 1c). Such a clock would entail a nonlinear, delayed negative feedback mechanism driving cell autonomous oscillations in gene expression (Novák and Tyson 2008). Through combining such a clock with a so-called morphogen wavefront that stops oscillations while memorizing phase, these temporal oscillations become translated into a spatially periodic pattern (for the original model, see Cooke and Zeeman 1976; for a detailed multi-scale model, see Hester et al. 2011; for recent models, see review by McDaniel et al. 2024). For somitogenesis, the molecular players underlying both clock and wavefront have been identified in considerable detail (see, eg Palmeirim et al. 1997; Dubrulle et al. 2001; Aulehla et al. 2003). For lateral root priming, a series of modeling studies has proposed experimentally observed negative feedback in auxin signaling to drive gene expression oscillations. These models involve negative feedback arising from either auxin-mediated freeing of ARFs inducing the expression of the ARF repressive AUX/IAA proteins (Middleton et al. 2010; Muraro et al. 2011, 2013) or ARF-mediated induction of auxin degradation (Mellor et al. 2016) and delays arising from slow ARF dimerization dynamics (Middleton et al. 2010).

The semantics and citations in the literature indicate that experimental plant scientists have embraced the concept of a cell autonomous root clock and largely ignore the potential for a more tissue-level emergent mechanism (see, eg Wachsman et al. 2020; Dickinson et al. 2021; Bustillo-Avendaño et al. 2022; Kircher and Schopfer 2023), with a notable exception being (Reyes-Hernández and Maizel 2023). This broad acceptance can be understood from the fact that other clocks, such as the cell cycle and circadian clock, are shared across kingdoms. Additionally, some striking similarities exist between the antagonistic FGF/Wnt and RA gradients along the vertebrate body axis and the auxin/PLT and cytokinin signaling gradients in the root tip (Ten Tusscher 2020). Still, an important question is whether similar mechanisms should be expected to be evolutionary selected for animal segmentation and root system branching. In animals, symmetry, scaling, and precision are of key importance for mobility and fitness. In plants, adaptability to environmental condition instead is essential (Ten Tusscher 2020). Additionally, there are also striking differences between the 2 processes. Somitogenesis entails oscillations in the growth zone and individual cells undergo multiple rounds of oscillations, while lateral root priming seemingly involves only one-half a round of an oscillation in the elongation zone where cells no longer divide (Traas and Vernoux 2010; Reyes-Hernández and Maizel 2023).

Importantly, none of the modeling studies investigating the potential for a root clock have shown auxin (signaling) oscillations to arise specifically in the elongation zone from the modeled auxin signaling network and local parameter conditions. Instead, the models by (Muraro et al. 2011, 2013) suggest that auxin promotes while cytokin inhibits oscillations, implying that oscillations would occur in the meristem and terminate in the early elongation zone (Dello Ioio et al. 2007; Salvi et al. 2020), contrasting with experimental observations. Indeed, in the only modeling study simulating oscillations restricted to the elongation zone, instead of oscillations endogenously emerging from the modeled regulatory interactions, oscillatory mRNA dynamics were locally imposed (Perianez-Rodriguez et al. 2021). While absence of proof can never serve as proof of absence, we can use mathematical modeling to investigate the (un)likelihood of a cell-autonomous root clock mechanism operating from the so-called oscillation zone region to generate the periodic priming dynamics observed in planta. Using simple models of auxin signaling, I demonstrate how in single cells oscillations occur only in a limited parameter domain that is further restricted by the interwinement of multiple AUX/IAA and ARF species into a single auxin signaling network and/or the addition of PIN mediated auxin export. Moving from cell to tissue level, I show that oscillations that start in the elongation zone cannot drive periodic prebranch site formation due to a lack of global oscillator phase memory. While periodic inputs such as periodic root cap apoptosis may introduce phase shifting, this would take several oscillation cycles to unfold; additionally the stimulus rather than the oscillator would be setting the pace. I conclude that an autonomous root clock operating from the oscillation zone is highly unlikely to underly lateral root priming.

## Results

### A primer on clocks, limit cycles, and oscillations

**Figure 2:**
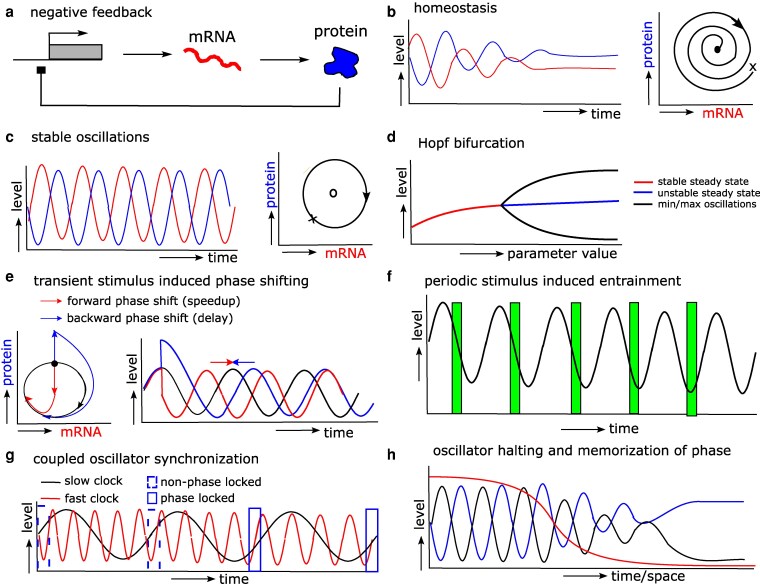
Homeostasis vs sustained oscillations, and oscillator modulation. a) A simple genetic negative feedback system in which the protein encoded by a gene negatively regulates its own expression. b) Example dynamics for a situation where negative feedback leads to homeostasis, the convergence of the system to a stable equilibrium, as a function of time (left) and in a 2D phase space (right). c) Example dynamics for a situation where negative feedback leads to stable oscillations, as a function of time (left) and in a 2D phase space (right). d) Bifurcation diagram showing how equilibrium and oscillation presence and stability change as a function of parameter value. Stable oscillations arise from a Hopf-bifurcation, involving a destabilization of a previously stable equilibrium. e) A single stimulus can shift the phase of an oscillator forward (red) or backwards (blue), causing the system to be ahead or behind of the state it would have been in without the stimulus. f) Repeated application of a stimulus (timing indicated by the green bars) results in the eventual alignment of a particular oscillator phase (here the valley) with the stimulus. g) Coupling of oscillators with substantially different periods results in adjustments of both oscillators resulting in eventual alignment of particular phases in both oscillators, in this case the upward phase of both oscillators. h) A oscillation impacting morphogen gradient (red line) through which individual cells (black and blue lines) sequentially traverse enables memorization of different oscillator phases in different cells. The memorization requires an additional mechanism besides the oscillator and morphogen gradient.

For oscillations to emerge in a biological system, a so-called negative feedback loop is essential (Lewis 2003; Novák and Tyson 2008). One of the simplest examples is shown in Fig. 2a, where a gene is translated into a mRNA that encodes a protein that acts as a repressive transcription factor for the expression of its own gene. Negative feedback in itself is insufficient to give rise to oscillations, instead the default behavior is homeostasis (Lewis 2003; Novák and Tyson 2008), (Fig. 2b).

Only if interactions are sufficiently temporally delayed and input-output relations are nonlinear (Lewis 2003; Novák and Tyson 2008), sustained, regular oscillations may arise (Fig. 2c). Nonlinearities allow amplification of protein levels that are only slightly above equilibrium into mRNA levels more strongly below equilibrium, causing the system to over- and undershoot. Delays cause mRNA levels to respond to earlier occurring protein levels rather than current protein production, further preventing the system to correct itself. Plotting these dynamics on a 2D mRNA vs protein plane, we see that for homeostasis the system spirals inward to a stable equilibrium (Fig. 2b, right), whereas for oscillations the system keeps walking over a series of connected points in this 2D plane (Fig. 2c, right) like the hands that keep traversing the face of a clock. In mathematical terms this path of connected points is called a limit cycle. Nonlinearities and delays frequently occur in biological systems. Nonlinearities, for example, arise from cooperative binding of the transcription factor protein to its promotor, resulting in a nonlinear translation of protein levels into gene expression changes. Delays arise from transcription, splicing, translation, and dimerization and transport of mRNA and protein between nucleus and cytosol (Novák and Tyson 2008). Finally, in addition to negative feedback, delay and nonlinearity, also other parameters affect oscillatory behavior. A parameter dependent transition of a system between stable and oscillatory behavior is called a Hopf bifurcation (Fig. 2d) (for an explanation of bifurcation diagrams, see Fig. S1).

### Control and coordination of clocks

While oscillators can autonomously maintain their cyclic behavior, they can be impacted by other factors. Phase shifting results from the effect of a single transient stimulus on 1 or more clock variables (Winfree 1980; Ermentrout and Terman 2010). In this case, I assume our stimulus either transiently enhances (Fig. 2e left, blue) or decreases (Fig. 2e left, red) protein level. In the 2D plane, we see how protein increase causes a detour while protein decrease causes a shortcut relative to the normal limit cycle (Fig. 2e left), resulting in a backward and forward phase shift, respectively (Fig. 2e, right). Thus, while oscillation period (the time it takes before the dynamics repeats itself) is unaffected, timing is. In contrast, entrainment results from the repeated, periodic application of a stimulus. Here, because the stimulus repeatedly pushes 1 or more of the systems variables in a certain direction, the system responds by aligning a particular oscillation phase with the timing of the stimulus (Fig. 2f, the oscillator aligns its valley with the green stimulus). A familiar example is the entrainment of organism's circadian clock with the light period during the day (Bell-Pedersen et al. 2005). Entrainment affects both phase and period, and if entrainment period differs more strongly from the free running period stronger stimuli are required (Winfree 1980; Ermentrout and Terman 2010).

Often oscillators are coupled to other oscillators, for example in multi-cellular tissue, where individual cells become synchronized through intercellular communication (Winfree 1980; Ermentrout and Terman 2010). Synchronization becomes less trivial for oscillators with a very different frequency (Fig. 2g)—for example, the cell cycle and circadian clock within a single cell. Here synchronization implies the adjustment of frequency and phase of both oscillators, causing them to become phase locked to one another in a particular phase (Winfree 1980; Ermentrout and Terman 2010). Similar to entrainment, this requires sufficiently strong coupling, while oscillator frequencies (inverse of period) should not deviate too far from a 1:n ratio (where n is an integer). Finally, as, for example, in the case of vertebrate somitogenesis, oscillations may need to terminate and be transduced into a stable spatially periodic pattern (Fig. 2h). This typically involves a gradual change in a parameter controlling oscillations, like the FGF/WNT morphogen gradient in somitogenesis. While terminating oscillations can be achieved by reducing delays, or by preventing the production of the repressive protein, an additional mechanism is required for memorizing oscillator phase prior to or coincident with full oscillator termination (see, eg François and Mochulska 2024).

### Auxin signaling drives oscillations within a constrained parameter range

**Figure 3:**
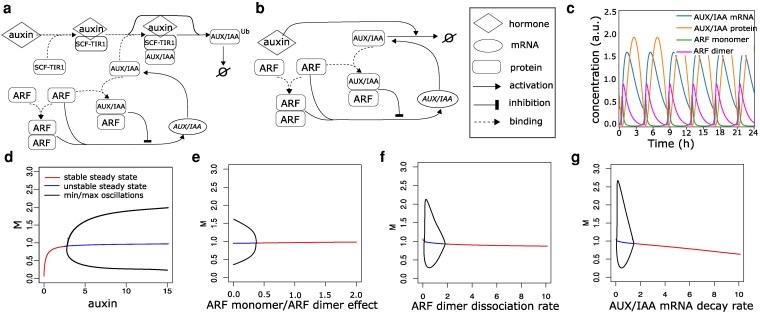
Oscillatory capacity of a single AUX/IAA single ARF system. a) Overview of the major players and interactions in plant auxin signaling as modeled in the Middleton et al. (2010) model. b) Overview of the simplified auxin signaling model in this work. c) oscillatory dynamics generated by the model shown in B for the parameter settings shown in Table S2. d–g) Bifurcation diagrams showing dependence of oscillatory dynamics on auxin level (d), the ratio of effectiveness in inducing AUX/IAA gene expression of ARF monomers vs dimers that affects non-linearity (e), the rate of ARF dimer dissociation that affects delay duration (f) and the AUX/IAA mRNA decay rate (g). In (d–g), except for the bifurcation parameter, parameters were identical to those in C.

The root clock underlying lateral root priming is assumed to arise from oscillations in auxin signaling and/or auxin levels. Canonical auxin signaling involves the binding of an auxin molecule to a single TIR/AFB receptor molecule (Kepinski and Leyser 2005), with the resulting auxin-TIR/AFB complex binding to an AUX/IAA protein (Kepinski and Leyser 2004), forming an auxin-TIR/AFB-AUX/IAA complex in which the AUX/IAA protein can be ubiquitinated, tagging it for degradation (Fig. 3a). Free non-ubiquitinated AUX/IAA protein can bind to auxin response factors (ARFs), preventing ARFs from inducing auxin dependent gene expression (Weijers et al. 2005). Auxin thus induces auxin dependent gene expression by derepressing ARFs. ARFs are found to homodimerize, with dimers being more potent inducers of gene expression (Ulmasov et al. 1999; Fontana et al. 2023). For at least a subset of AUX/IAA genes, ARFs induce expression of these genes, resulting in a negative feedback loop (Middleton et al. 2010; Vernoux et al. 2011).

Several similar models of the auxin signaling system have been made (Middleton et al. 2010; Vernoux et al. 2011), and here I developed a simplified version of the Middleton 2010 model (Fig. 3b). Briefly, the number of variables was reduced from 9 to 5 by transforming auxin into a control parameter, assuming instantaneous degradation of ubiquitinated AUX/IAA and using so-called quasi-steady state assumptions for auxin, TIR, and AUX/IAA interactions, while maintaining oscillations under largely similar parameter conditions (for details, see Methods). As for the original model, for a specific range of parameter conditions, that is, parameter values such as auxin levels, transcription rates, and affinities are constrained between certain minimum and maximum values, oscillatory dynamics can be obtained (Fig. 3c). Still in addition to reductions in auxin levels (Fig. 3d), reductions in nonlinearity (the size difference in effectiveness of ARF dimers vs monomers on transcription), temporal delays in the negative feedback loop (dimer dynamics), and differences between AUX/IAA mRNA versus protein turnover readily transform oscillatory into steady-state dynamics (Fig. 3e–g). These results are consistent with general oscillator theory (Novák and Tyson 2008). Indeed, in the strongly related Vernoux et al. (2011) model, which includes AUX/IAA rather than ARF dimerization, the authors failed to identify parameter settings supporting oscillations. Thus, in a single ARF, single AUX/IAA system oscillations occur in a limited parameter domain that may not trivially occur in planta.

### A 2 ARF-2 AUX/IAA system further constrains oscillation capacity

**Figure 4:**
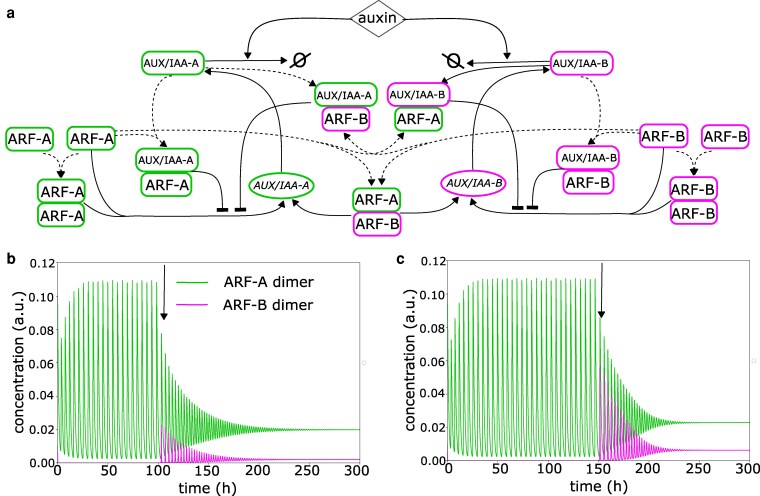
Termination of oscillations in two AUX/IAA 2 ARF systems. a) Architecture of two AUX/IAA 2 ARF auxin signaling network in which both AUX/IAAs can dimerize with both ARFs and ARFs can heterodimerize. The “A”-pair (indicated in green) is parametrized to support oscillations (Table S2), the “B”-pair (indicated in red) is parameterized to not support oscillations (Table S2) b) System dynamics during an initial phase in which only the “A” pair is expressed, and a second fase (start indicated by the arrow) where also the “B” pair is expressed. c) Same as in B but now with ARF-A monomers and dimers having 20-fold less affinity for AUX/IAA-B promotors than ARF-B monomers and dimers, and ARF-B monomers and dimers having 20-fold less affinity for AUX/IAA-A promotors than ARF-A monomers and dimers. ARF heterodimers have 20-fold lower affinity for both AUX/IAA promotors.

Importantly, Arabidopsis contains a total of 23 ARFs and a total of 29 AUX/IAAs, and in most plant tissues a combination of several ARFs and AUX/IAAs are simultaneously present (Weijers et al. 2005; Rademacher et al. 2011; Vernoux et al. 2011). To investigate the impact on oscillatory dynamics, I considered a 2 ARF-2 AUX/IAA system, assuming that the different ARFs could heterodimerize with one another, as well as with both AUX/IAAs (Fig. 4a). I start from a situation in which only 1 ARF AUX/IAA pair is expressed (green, A), parametrized such that dynamics are well within the oscillatory regime (Table S2). After reaching stable oscillations, I allow expression of the second ARF AUX/IAA pair (red, B) parametrized to not oscillate (Table S2). If maximum AUX/IAA and ARF protein levels of pair B are 25% or more than of pair A, oscillations were halted (Fig. 4b).

Above, the 2 ARFs and AUX/IAAs only differed in turnover and dimerization rates, thereby affecting their potential to support oscillations. Additionally, ARFs and AUX/IAAs may differ in binding affinity and effects on target genes. Therefore, I redid the above simulation, with ARF A monomers and dimers mostly affecting expression of AUX/IAA A, and ARF B mostly affecting expression of AUX/IAA B through implementing a 20-fold difference in the binding affinities of ARFs to the different AUX/IAA promotors. Keeping all else identical, the ARF-AUX/IAA B pair now needed to be expressed at at least 40% of the ARF-AUX/IAA A pair to halt oscillations (Fig. 4c). Thus, even with a partial separation between the 2 pairs, oscillations can be easily terminated through the addition of a second, numerically nondominant non-oscillatory ARF-AUX/IAA pair.

This indicates, that in planta, with multiple AUX/IAAs and ARFs, parameter constraints need to at least be partially met by all AUX/IAAs ARFS in the oscillation zone to support oscillations. This further underlines the nontriviality of meeting these conditions.

### A degradation and import based auxin signaling oscillator can only function in absence of significant auxin export

**Figure 5:**
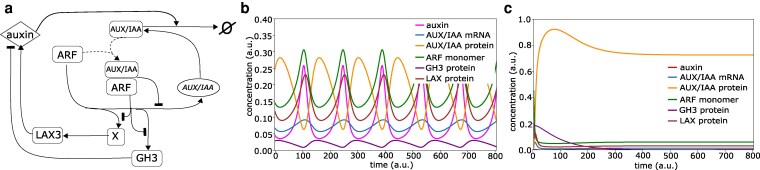
Termination of oscillations in a degradation-import oscillator by PIN efflux. a) Interaction network of auxin signalling, GH3 mediated degradation and LAX3 mediated import as modeled in Mellor et al., (2016). b) Oscillatory dynamics resulting from the parameter settings described in Table S3. c) Non-oscillatory dynamics resulting from the same parameter settings but adding a small PIN mediated auxin efflux term.

In an effort to overcome the parameter constraints on cell autonomous auxin signaling oscillations, expanded models have been proposed where delays and nonlinearity no longer depend on ARF-dimer–dominated induction of AUX/IAA gene expression but arise from ARF-induced LAX3 and GH3 expression (Mellor et al. 2016). Negative feedback arises from GH3 degrading auxin, while counteracting positive feedback arises from the auxin importer LAX3 (Fig. 5a). Combining negative feedback with positive feedback is a well-known method to achieve oscillations (Novák and Tyson 2008). In isolated cells under constant external auxin conditions and specific parameters, this model will result in sustained oscillations (Fig. 5b, Table S3) (see also Mellor et al. 2016). Note that here oscillations in auxin signaling necessarily involve oscillations in auxin concentration (Fig. 5b). Importantly, pericycle and protoxylem cells, where priming is assumed to occur, express polarly localized auxin exporting PIN proteins. Even when assuming PIN expression levels to be 10% of those of LAX levels, and have 70-fold lower auxin conductance, incorporation of PINs abolishes oscillations (Fig. 5c). Thus, in a tissue context, oscillations can only arise in regions where overall tissue level transport supports sufficiently high auxin concentrations. Consequently, these oscillations are not cell autonomous.

### A model for tissue-level evaluation of the root clock hypothesis

**Figure 6:**
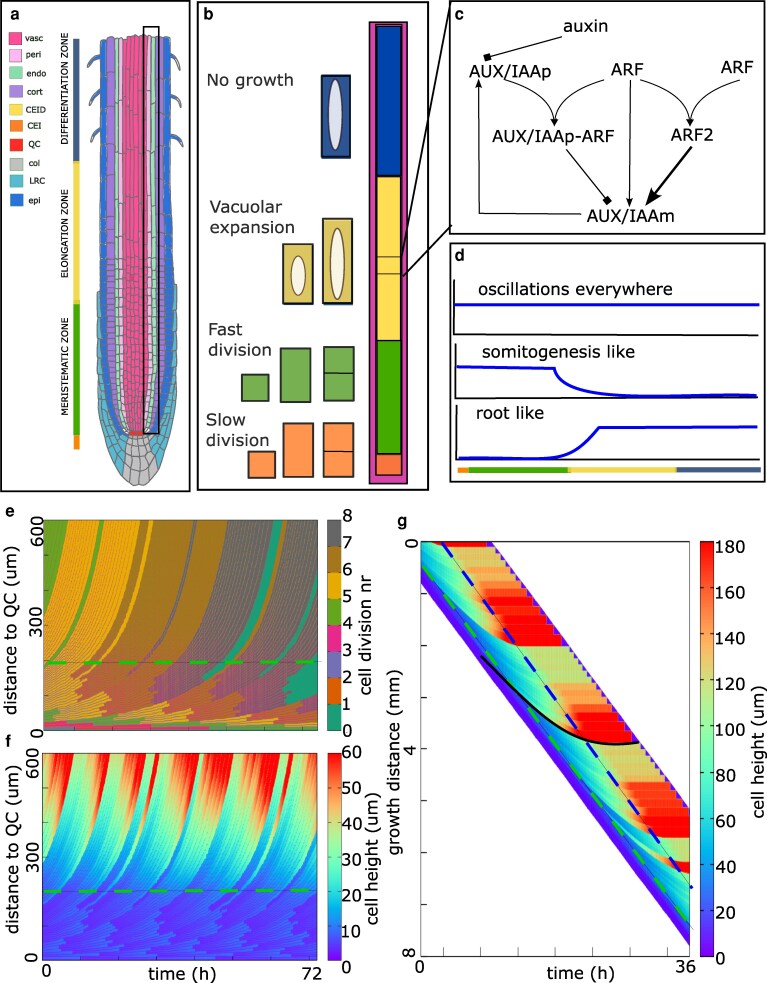
1D growing tissue model with oscillatory network. a) Overview of Arabidopsis root tip anatomy with the different cell types organized largely in cylindrical fashion and the different longitudinally organized developmental zones. Pericycle and nearby protoxylem cell files are indicated by the boxed in area. b) Layout of the 1D tissue model with developmental zones and corresponding growth dynamics. c) Simplified representation of the auxin signaling network incorporated in all individual cells. d) Possible parameter gradients (blue) governing where oscillations can occur (high level) and cannot occur (low level). e, f) Cell dynamics kymograph where cells are colored based on the number of cell divisions they underwent (e) or their cell height (f) g) Cell size dynamics kymograph from the same model simulation as in F but now showing a larger domain of the simulated root and with the root tip following actual growth trajectory instead of its position aligned to the horizontal axis. The black line indicates the trajectory of an individual cell. Dashed green lines indicate the top boundary of the meristem, dashed blue lines indicate the top boundary of the elongation zone.

Independent of possible parameter constraints on cell-autonomous oscillatory dynamics, a major unexplored question is whether elongation zone–localized cell-autonomous oscillations can drive priming dynamics. To investigate this, I simulate a 1D tissue representing a single protoxylem and/or pericycle pole (Fig. 6a), in which each cell contains the auxin signaling network (Fig. 6c) and in which cells in the meristematic zone (MZ) divide, in the transition zone (TZ) slowly grow and in the elongation zone (EZ) rapidly expand (Fig. 6b). In the differentiation zone, cell expansion has terminated and cells maintain their size. By varying parameter settings along the 1D tissue strand, specifically the level of ARF, one can influence where in the tissue oscillations occur (Fig. 6d). In addition to the auxin signaling network, a simplified memorization mechanism is implemented that beyond a certain point in the tissue and within a limited spatial window memorizes the state of the auxin signaling network to enable transformation of temporal oscillations into spatial stripes, that is, prebranch sites (PBS). Cell growth, division and expansion rates, meristem size, and differentiation rates were fitted to reproduce recent detailed growth tracking data from Goh et al. (2023) (compare Figs 6e–f to 4a in Goh et al. 2023) (see Methods, Table S4). Of note, given the focus on cell-autonomous rather than tissue-level emergent oscillations, feedback between auxin and auxin transport and/or growth dynamics was ignored. In the model a larger region of the root is simulated than tracked in the Goh et al. experiments (2.5 mm vs 600 microm), allowing one to further track cell size variations, growth dynamics, and PBS formation into the differentiation zone, for which typically downward oriented kymographs are used (Fig. 6g).

### MZ localized oscillations can drive periodic PBS formation

**Figure 7:**
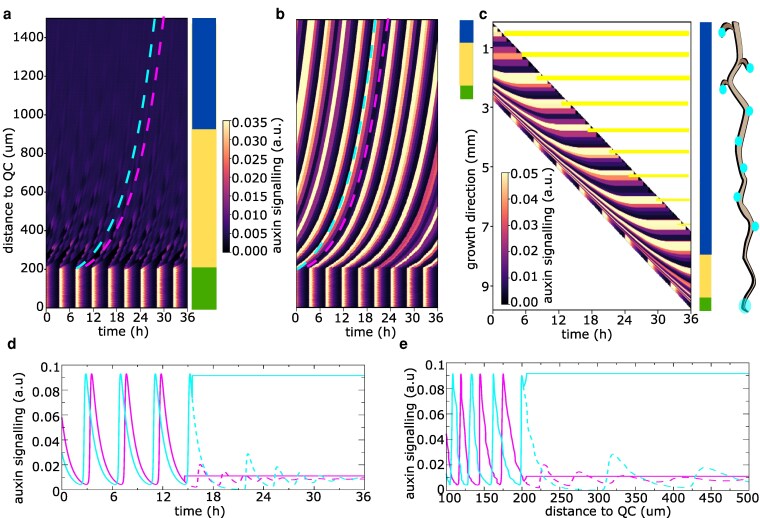
MZ driven oscillations periodically patterned PBS. a) Kymograph depicting auxin signaling dynamics (ARF dimer level) for a situation where the parameter controlling the total amount of ARF in the system is lowered from MZ to EZ such that oscillatory dynamics are only supported in the MZ. For ease of orientation the root zones to which the different distances from the QC correspond are depicted. b) Kymograph depicting auxin signaling dynamics (MZ) and subsequent memorization (from start of EZ upwards). c) Same kymograph as in B, but now for root tip displacing downward during growth. Again, orientation root zones are indicated. Furthermore, yellow lines indicate how high auxin peaks would continue if the simulation domain kept growing like a real root instead of having a constant finite size, with the root on the right indicating how this would result in PBS and lateral root primordia patterning. d) Auxin signaling dynamics in 2 separate cells (cyan and green lines), in absence (dashed lines) and presence (continuous lines) of memorization dynamics, as a function of time. Trajectories of these cells are indicated in A and B. e) Same dynamics as in D but now as a function of distance from QC.

To investigate the relevance of the location in which cell-autonomous oscillations occur, I first applied a gradient in the level of ARF that is high in the meristem and low in the elongation and differentiation zone that ensures that oscillations are supported in the meristematic growth zone (Fig. 6d middle pannel). Note that this situation resembles the situation in vertebrate somitogenesis and arthropod clock-driven segmentation where oscillations occur in the posterior growth zone and terminate anteriorly. For the simplified model, parameters were chosen such that for the high oscillation supporting ARF levels an oscillation period of approximately 4.2 hours arose, in the range of experimentally reported values (De Smet et al. 2007; Moreno-Risueno et al. 2010; Xuan et al. 2015). One can see a clear temporal activity pattern arises in the meristem, indicating that all meristematic cells are oscillating in phase (Fig. 7a). Upon entering the elongation zone, these oscillations dampen and terminate (Fig. 7a). After adding a mechanism memorizing auxin signaling state at the start of the elongation zone, this transient temporal oscillatory pattern becomes transduced into a spatially periodic pattern in the rest of the root (Fig. 7b–c). To further illustrate this, Fig. 7d and e show dynamics of 2 individual cells, their trajectories indicated in Fig. 7a and b, as a function of either time or distance. The figures compare non-memorized (dashed lines) with memorized dynamics (solid lines), again showing how different cells enter the elongation zone sequentially and hence at different phases (Fig. 7d), different memorized auxin signaling states ensue. This enables periodic patterning of PBS, as observed in experiments.

Importantly, in planta, oscillatory activity related to priming has only been observed either just above the meristem (De Smet et al. 2007; De Gernier et al. 2025) or higher up in the elongation zone (Moreno-Risueno et al. 2010; Xuan et al. 2015, 2016; De Gernier et al. 2025), depending on the reporter used, but not inside the meristem itself.

### Above MZ driven oscillations cannot periodically pattern PBS

**Figure 8:**
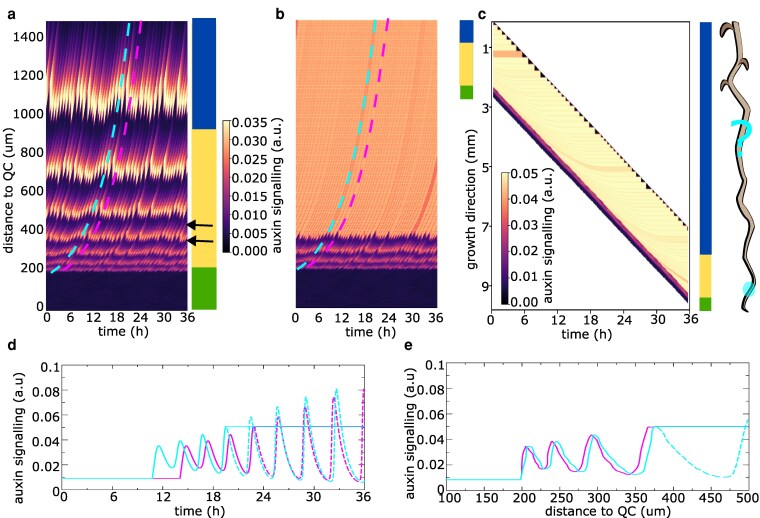
EZ localized oscillations result in spatial synchronization. a) Kymograph depicting auxin signaling dynamics (ARF dimer level) for a situation where the parameter controlling the total amount of ARF in the system is increased from MZ to EZ such that oscillatory dynamics are only supported in the EZ and above. b) Kymograph depicting auxin signaling dynamics (MZ) and subsequent memorization (from halfway EZ upwards, bottom arrow in A). c) Same kymograph as in B, but now for root tip displacing downward during growth. d) Auxin signaling dynamics in 2 separate cells (cyan and green lines), in absence (dashed lines) and presence (continuous lines) of memorization dynamics, as a function of time. e) Same dynamics as in D but now as function of distance from QC.

**Figure 9:**
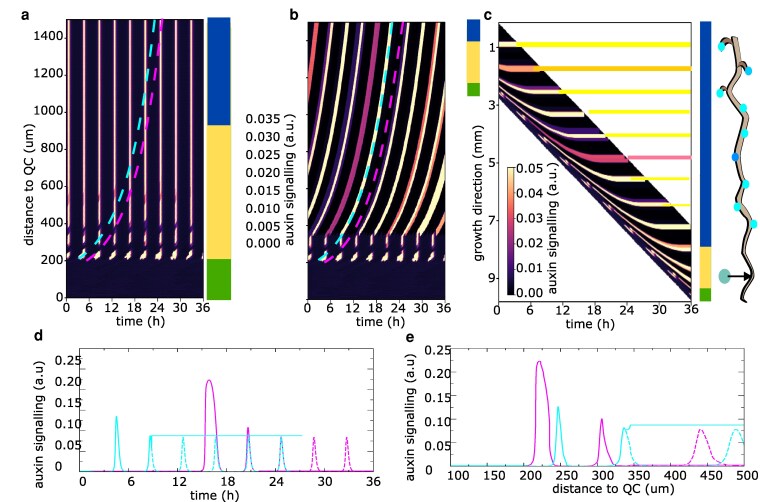
Superimposed AUX/IAA oscillations in EZ drive periodic PBS formation. a) Kymograph depicting auxin signaling dynamics (ARF dimer level) for a situation where AUX/IAA expression dynamics are superimposed to be sinusoidal, and total amount of ARF does not support autonomous oscillations. In the root on the side illustrating the eventual result in PBS the oscillatory zone is put aside the root, to indicate it's superimposed rather than endogenous nature. b) Kymograph depicting auxin signaling dynamics (MZ, early EZ) and subsequent memorization (from halfway EZ upwards. c) Same kymograph as in B, but now for root tip displacing downward during growth. d) Auxin signaling dynamics in 2 separate cells (cyan and green lines), in absence (dashed lines) and presence (continuous lines) of memorization dynamics, as a function of time. e) Same dynamics as in D but now as function of distance from QC.

Next, I implemented “root-like” conditions, where oscillatory dynamics are only supported above the meristem by implementing low ARF levels in the MZ and higher ARF levels in the EZ and DZ (Fig. 6d bottom panel). Consistent with this, oscillations start in the elongation zone, with cells that at different time points reside at the same physical distance from the QC displaying the same oscillation phase, resulting in a horizontally banded spatial pattern in the (unmemorized) kymograph (Fig. 8a). Observed small variations in the horizontal direction arise from stochasticity in cell growth and division causing minor timing differences in when a cell enters the elongation zone. This dramatically different behavior arises from the fact that while individual cells still oscillate, they now all enter the elongation zone from the same non-oscillatory condition, starting oscillations in the same phase (Figs 8d and 9e). Applying the memorization mechanism approximately halfway through the elongation zone, either all cells end in a high (Fig. 8b) or low auxin signaling state (not shown), depending on the exact positioning of the memorization (bottom vs top arrow in Fig. 8a). Importantly, these results do not critically depend on all cells entering the elongation zone with exactly the same state. To illustrate this point additional simulations incorporating stochasticity in dividing maternal auxin signaling factors over daughter cells after division (Fig. S2a), gene expression noise (Fig. S2b), or noise in the auxin level (Fig. S2c) were performed. While these introduce small state differences between cells entering the EZ, these do not lead to significant, systematic phase differences needed to periodically separate PBS from non-PBS forming cells.

These results reveal a fundamental problem with the applicability of the somitogenesis clock mechanism to plant roots. In somitogenesis and our simulation with meristem localized oscillations, oscillations occur in the stem cell niche and division zone. Assuming symmetric division of auxin signaling factors over daughter cells, 2 new cells inherit their oscillatory state from their mother cell. As a consequence, in the stem cell niche where divisions both maintain the stem cell pool and produce novel transit amplifying cells, there is the potential to maintain the oscillation history and pass on the current phase of the cycle to new cells. As a consequence, there is tissue level time keeping and cells sequentially entering the EZ do so with different phases (Fig. S3a). Importantly, this potential for time keeping is maintained if we increase model realism by incorporating cell division, gene expression, or auxin input noise (Fig. S4). In contrast, if as observed in planta oscillations start only in the EZ zone, no tissue-level timekeeping exists and instead all cells start oscillating from a highly similar state and phase as if pressing their stopwatch when passing the EZ start mark (Fig. S3a). These results follow from general oscillator dynamics, independent of oscillator details, cell-cell coupling, gradients or noise, with cells undergoing the Hopf bifurcation at the same position.

### Global oscillator input can drive periodic PBS formation

The above may explain why in the Perianez-Rodriguez et al. (2021) study, which simulated oscillations specifically in the early elongation zone, oscillatory mRNA dynamics needed to be superimposed in this zone. To validate this hypothesis, I took a similar approach, using non-oscillatory parameter conditions (Table S2) and replacing ARF mediated IAA transcription with superimposed oscillatory transcription (equation 20b Methods). Our model output indicates that periodic elevations in auxin signaling in the early elongation zone now could indeed be obtained (Fig. 9a). Without a memorization mechanism, oscillations continue and individual cells undergo multiple rounds of oscillations (Fig. 9a, d, e), while adding a memorization mechanism allows cells arriving at different phases of the global oscillator to generate a spatially periodic pattern (Fig. 9b–e).

Importantly, by superimposing IAA mRNA oscillations, I effectively globally dictated oscillations rather than allowing them to naturally emerge from the interactions in the auxin signaling network. Note that it would be entirely unclear where this global oscillation phase information would be stored, given that individual cells do not persistently reside in the EZ. Additionally, by superimposing IAA mRNA oscillations one is not simply providing a periodic input signal to the root clock but rather magically driving the expression of 1 of its genes.

### Limited rescue of periodic patterning from periodic sources

Next, I therefore investigated to what extent periodic input could enable periodic variations in the elongation zone. Because cells only start oscillating in the EZ and it is the first peak/valley that is being transduced to a PBS/no PBS, time is insufficient for entrainment (Fig. 2f, g); periodic signals can only provide a single-phase shifting input (Fig. 2e). Therefore, I considered periodic processes that operate at periods close to the priming period near the start of the EZ. Candidate mechanisms are root cap apoptosis resulting in export of auxin from the root cap to the rest of the root (Xuan et al. 2015; De Gernier et al. 2025) observed to occur with a periodicity of 4 to 5 h (Fendrych et al. 2014; Xuan et al. 2015), periodic changes in elongation zone cell sizes and hence auxin uptake potential (van den Berg et al. 2021), and possibly cell cycle state given the recent discovery of faster cell cycles in the top part of the meristem (Echevarria et al. 2025). Additionally, periodic phloem unloading could be a candidate mechanism, but so far this has been reported to occur at far higher frequencies (Ross-Elliott et al. 2017). I therefore tested whether a periodic variation in auxin input in the bottom 20% of the elongation zone, spanning approximately 3 to 4 early elongation zone cells, could drive variations in oscillator phase in the elongation zone (equation 41 Methods).

**Figure 10:**
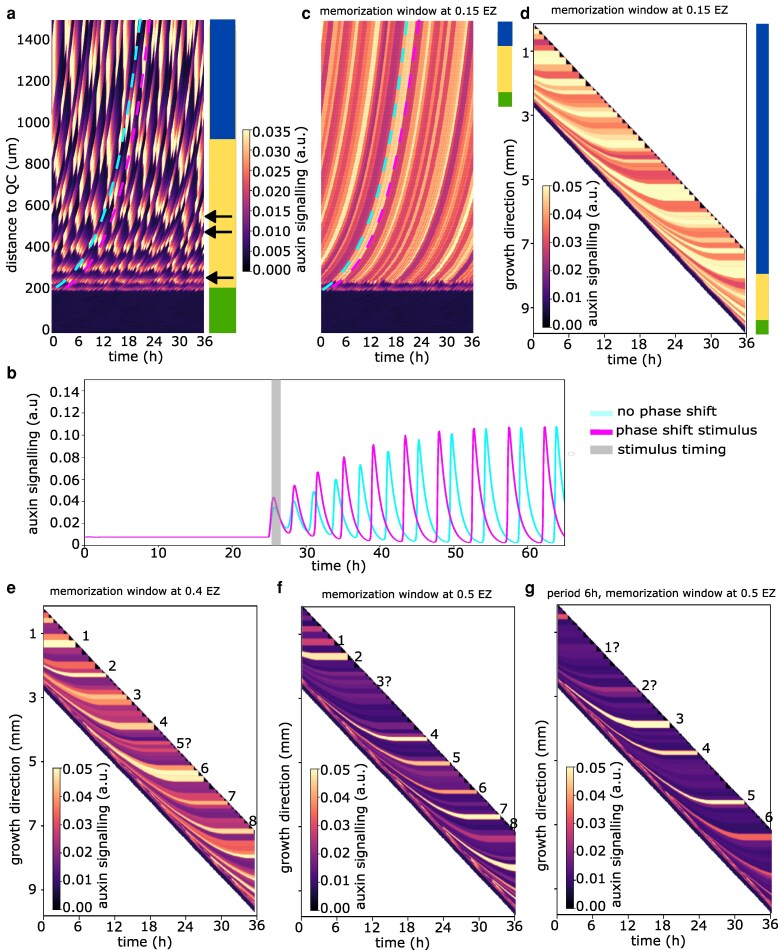
Periodic auxin input to EZ localized oscillations result in phase shifting. a) Kymograph depicting auxin signaling dynamics (ARF dimer level) for a situation where a 4.2 h periodic increase of auxin is imposed in the early EZ (bottom 20%) and the parameter controlling the total amount of ARF in the system is increased from MZ to EZ such that oscillatory dynamics are only supported in the EZ and above. b) Temporal development of auxin signaling dynamics in a cell exposed to the auxin stimulus (cyan) and a cell not exposed to the auxin stimulus (green), with the timing of the auxin stimulus for the cyan cell indicated with the grey area. c) Kymograph depicting auxin signaling dynamics and subsequent memorization from a position at 0.15 EZ (bottom arrow in A). d) Same kymograph as in B, but now for root tip displacing downward during growth. e) Downard oriented kymograph for situation where memorization occurs from a position at 0.4 EZ (middle arrow in A). f) Downward oriented kymograph when memorisatin occurs from 0.5 EZ (top arrow in A). g) Downward oriented kymograph for a situation where auxin input is applied with a 6 h instead of 4.2 h period, with memorization at 0.5 EZ. For E-G we indicated the hypothetical PBS, taken as elevations in memorized auxin signaling state with numbers. Question marks are added if signaling level is less clearly elevated, likely resulting in unstable PBS.

Figure 10a shows a kymograph when providing an influx of auxin at a frequency close to the frequency of the auxin signaling driven oscillations (4.2-h period). Comparing along the horizontal direction whether changes in activity occur, we see that at positions just above the meristem hardly differences occur, while higher up groups of cells become more out of phase. Tracking oscillation dynamics in individual cells (Fig. 10b), we indeed see that while the auxin stimulus has an instantaneous effect on auxin signaling levels (grey area in Fig. 10b), phase shifting takes several cycles to unfold. While I cannot exclude the existence of stimulus parameters for which substantial phase shifting occurs instantaneously, this delayed unfolding of a phase shift is a well-known property of nonlinear oscillators (Winfree 1980; Kuramoto 2003), making this highly unlikely. As a consequence, if memorization of auxin signaling state occurs close to the meristem (bottom arrow in Fig. 10a), memorized states show limited differences between cells (Fig. 10c–d) making it hard to distinguish prebranch from nonprebranch sites. If memorization occurs later (middle and top arrows in Fig. 10a), once several cycles of oscillations have occurred and phase differences have developed this distinction becomes clearer (Fig. 10e–f), and PBS numbers align with the number of stimuli fitting within the time window (approximately 8 for a 4.2-h period in 36 h). Note that depending on the exact memorization position, different cells will memorize a high auxin signaling state (compare Fig. 10e and f), and patterning is irregular in both spacing and amplitude.

Figure 10g shows auxin signaling and memorization if an auxin stimulus period of 6 h, slower than the inherent oscillator frequency, is used. The kymograph now shows 6 instead of 8 memorized high auxin peaks, indicating that stimulus frequency rather than endogenous oscillator frequency dictates PBS formation rate. This begs the question of what then the oscillator itself is for, although a role in translating signal modality or amplitude could be possible. Combined these results indicate that adding a periodic phase shifting signal to a EZ localized cell autonomous oscillator would result in a nonparsimonious, nonrobust mechanism that furthermore requires multiple rounds of oscillations, inconsistent with experimental observations.

### Cryptic meristem oscillations are inconsistent with experimental data

A final seemingly logical hypothesis would be to propose the existence of oscillations in the meristem that have not yet been picked up experimentally, which through time transform into the observed oscillations in the EZ. This would solve the problem of global time keeping and allow for differential phases in which cells arrive at the EZ. Indeed, for zebrafish somitogenesis oscillations have been observed to increase in amplitude and decrease in frequency before their transformation into a temporally stable pattern (Shih et al. 2015). Thus, the question becomes what parameter change could explain the transition from unnoticed MZ to clear EZ oscillations. Note that this requires parameters that affect oscillation amplitude rapidly, given that only a single oscillation is observed in EZ and before no oscillations are observed.

To investigate this, I varied all 20 model parameters between a low and high amplitude generating value, that is the lowest value still generating sustained oscillations and a value at or close to the default oscillatory settings value (Table S2). For each parameter I recorded mean auxin signaling (ARF dimer) level for both the high and low amplitude value, the number of cycles needed to reach 95% of the new higher amplitude, the immediate and long-term amplitude and the change in oscillation period (Table S5).

For most parameters, amplitude increase requires a substantial number of cycles to fully unfold. Only for 3 parameters ($\alpha_{TIR}$, $p_{m}$, and $d_{auxin}$), oscillation amplitude overshot and then quickly equilibrated, allowing for a substantial part of the eventual amplitude increase to occur in the first cycle. Next, I assume that for oscillations to be hidden in the MZ while being clearly observable in the EZ, and at least 4-fold increase in amplitude is required. Only 2 ($b_{m}$ and $p_{m}$ controlling AUX/IAA levels) result in such an amplitude increase. For $p_{m}$, the only parameter satisfying both conditions, oscillations speed up instead of showing the required slowing down. Additionally, the transition from low to high amplitude oscillations involves a more than 2-fold increase in auxin signaling levels from the MZ to the EZ, inconsistent with experimental observations localizing the auxin signaling maximum in the MZ.

As an alternative hypothesis, a cryptic MZ and early EZ localized, non-auxin signaling based oscillator may exist that through an unknown mechanism drives auxin signaling in the EZ but not MZ. Building such an oscillator into the model (see Methods) shows that this can indeed drive out of phase auxin signaling oscillations in the EZ. However, for these oscillations to be of significant enough amplitude to reasonably enable the distinction between PBS and non-PBS sites, the impact of this cryptic oscillator on AUX/IAA expression should be of a similar strength as that of ARF-mediated expression (Fig. S5). Despite this, it should have thus far escaped our attention.

Summarizing, these results indicate that the existence of a cryptic MZ oscillator driving EZ oscillations, auxin signaling based or not, is very unlikely.

## Discussion

Molecular clocks are an important method for time keeping in biological organisms, with the most well studied examples being the cell cycle and circadian clock present in single and multicellular organisms across the kingdoms of life. Another well studied example from developmental biology is the so-called somitogenesis clock, in which temporal oscillations in gene expression through growth become translated into a spatially periodic pattern of gene expression prepatterning future somite halves and boundaries. Hallmarks of clocks are their autonomous persistent oscillatory behavior in absence of external inputs, their potential for phase shifting and entrainment by external inputs and their potential to synchronize with other oscillators. Based on the observation of periodic gene expression variation, a root clock akin to the somitogenesis clock has been proposed to underly lateral root priming (Moreno-Risueno et al., 2010). Still not all biological time keeping or sequentially ordered processes involve a clock, with a prominent example being the process of phyllotaxis, where auxin maxima prepatterning leaf primorodia are periodically formed from the interplay between tissue growth and auxin transport dynamics (Jönsson et al. 2006; Smith et al. 2006; Sassi and Vernoux 2013).

Indeed, several differences between somitogenesis and lateral root priming beg the question of whether a cell-autonomous root clock is involved in the latter. Firstly, in the root cells seemingly undergo only a half “oscillation” -either an upstroke or downstroke- in contrast to the several rounds of oscillations that cells undergo in somitogenesis and that play an important role in somite patterning (McDaniel et al. 2024). Additionally, while in somitogenesis oscillations occur in the posterior growth region, in lateral root priming oscillations occur only once cells have left the meristem. It may in fact be more likely that lateral root priming and prebranch site formation resemble plant phyllotaxis rather than somitogenesis, with phyllotaxis also producing periodic auxin maxima at regular distances from the meristematic cells (Jönsson et al. 2006; Smith et al. 2006; Sassi and Vernoux 2013). Note that also in phyllotaxis it would be expected that a constant distance from SAM, periodic variations in gene expression will arise despite the absence of oscillations in single cells.

Other evolutionary considerations may also argue against the likelihood of a root clock. First, in animals, mobility is of critical importance for survival and therefore a robust, symmetric and proportional body plan is essential, explaining the evolution of a clockwork type mechanism. In contrast, in immobile plants flexible adjustment to environmental conditions rather than stereotypical patterning is of key importance to survive in a variety of different conditions. Thus, selective forces acting on animal body axis segmentation vs root system branching are likely very different (K. Ten Tusscher 2020). Secondly, in animals with a segmented body plan the preferred solution appears to be a clock-and-wavefront type of segmentation, which likely has evolved in parallel not only in vertebrates but also in arthropods and annelids (François et al. 2007; Chipman 2010; Ten Tusscher and Hogeweg 2011). The simultaneous, hierarchical Drosophila segmentation is generally accepted as an evolutionary derived mode. In contrast, in plants expansion of the root system likely evolved from the bifurcating pattern observed in Lycophytes (Hetherington et al. 2020), and possibly via the merophyte based pattern in Ferns (Motte and Beeckman 2019). Finally, in Cucurbites, lateral roots still arise in the meristem (Ilina et al. 2018) as in evolutionary older plant lineages yet follow otherwise an Arabidopsis type route with founder cell specification. Thus, in the plant kingdom clearly no repeatedly evolved and subsequently well conserved elongation zone oscillatory mechanism is present.

This current study provides yet another strong argument against the likelihood of a cell autonomous root clock underlying lateral root priming. First, our study highlights the strong constraints on parameter conditions that apply for auxin signaling oscillations to occur, constraints that are further enhanced due to the presence of multiple AUX/IAA and ARF types typically being present in any plant tissue, as well as the presence of auxin exporters. It can of course be argued that in planta auxin signaling mechanisms are far more complex than those modeled here, and hence are likely to contain feedback mechanisms that can keep the system within the required constrained parameter domain. As a contra-argument, the 2015 Xuan et al. paper on the effect of root cap IBA to IAA conversion shows that oscillation period remains remarkably constant despite a 2.5-fold difference in oscillation amplitude. The large change in oscillation amplitude and hence auxin levels suggest that even in absence of normal buffering mechanisms, oscillation dynamics and frequency remain robust. This is inconsistent not only with the simplified models in this study but also with general nonlinear oscillator theory which shows that typically amplitude and frequency are hard to decouple. This further argues against the likelihood of oscillations arising from a cell-autonomous auxin signaling oscillator.

Irrespective of the plausibility of cell-autonomous auxin signaling oscillations, our study suggests that cell-autonomous oscillations occurring in the (early) elongation zone are highly unlikely to underly lateral root priming. A critical difference between somitogenesis and lateral root priming identified here is the region in which oscillations occur. In the region where stem cell divisions and hence inheritance of cell states occur a global system level memory for oscillator phase can be established. In a zone without stem cell division, cells transition from a non-oscillatory to a oscillatory state from largely identical conditions, causing them to all start oscillations with the same phase and status and prohibiting variations in activity in the early elongation zone. Our study furthermore indicates that even with additional periodic input into the root clock specifically at the elongation zone, which would present a highly non-parsimonious mechanism, generating sufficient variation in early elongation zone activity is highly nontrivial. Finally, we show that the presence of a hidden oscillator in the meristem that only becomes apparent in the elongation zone is unlikely. In contrast, the reflux-and-growth mechanism we have proposed previously offers a parsimonious explanation for why oscillations occur specifically in the early elongation zone and in narrow vasculature cells (Van den Berg et al. 2021). Finally, on the level of entire root system architecture, we recently developed models simulating overall root system architecture development to investigate the causes of differential root branching in brassinosteroid mutants (Khandal et al. 2025). In this study we demonstrated that growth based priming mechanisms such as reflux-and-growth or a Turing type mechanism could reproduce the reduced branching of brassinosteroid mutants. In contrast, a root clock mechanism, in which clock period is independent of root growth rate, would predict a highly branched architecture for brassinosteroid mutants, opposite to experimental observations.

I suggest that the previously reported periodic variations in gene expression (Moreno-Risueno et al. 2010) that have been interpreted as part of or downstream of a cell autonomous genetic oscillator could in fact all be downstream of an emergent, non-cell autonomous process producing periodic variations in auxin signaling. Previously proposed candidate mechanisms were the periodic root cap apoptosis, reflected flow, as well as the reflux-and-growth mechanism (Mironova et al. 2010; Xuan et al. 2015; van den Berg et al. 2021). Indeed, the fact that the periodic gene expression previously reported appears to consist of a sequence of waves containing different sets of genes appears more in line with a sequence of processes leading up to prebranch site formation set in motion by a periodic priming mechanism than that these gene expression waves are processes being part of the priming mechanism itself (Moreno-Risueno et al. 2010). Importantly, mutants in oscillating genes with substantially reduced prebranch site numbers, such as SHATTERPROOF1 and 2, have been taken as evidence of the existence of an oscillator circuit (Moreno-Risueno et al. 2010). However, no data on oscillation dynamics has been recorded for these mutants, leaving open the question whether oscillations per se are affected, or rather oscillation amplitude and hence the success rate of stable prebranch site formation. Previous studies reporting mutants in PBS formation capacity were related to cell wall mechanics and/or cell expansion (Wachsman et al. 2020; Dickinson et al. 2021) processes known to affect oscillation amplitude and PBS formation success according to the reflux-and-growth mechanism.SHATTERPROOF genes may similarly affect these processes given their role in fruit cell wall mechanics (Liljegren et al. 2000) and effects on auxin levels (Verma et al. 2025), yet these roles also open up potential feedbacks into a clock mechanism. Thus, without clarity on the effect of shatterproof mutations on oscillation frequency vs amplitudes and downstream gene expression, observed reductions in LRs can neither be used as support for or against an autonomous root clock. Finally, I suggest that the negative feedback mechanism recently reported by Perianez-Rodriguez and co-workers (Perianez-Rodriguez et al. 2021) is not negative feedback giving rise to oscillations, but rather negative feedback critical for auxin level homeostasis, which is necessary to produce regular spacing of prebranch sites. This is supported by their own modeling findings showing how in absence of this negative feedback an elevation of overall auxin signaling makes it hard to distinguish peaks and valleys in auxin signaling and hence results in precocious prebranch site formation. This is consistent with the previously reported importance of an auxin signaling minimum in the lateral root forming region (Dubrovsky et al. 2011).

## Methods

### Single cell models

#### Original Middleton 2010 model

The original Middleton et al. (2010) model describes the dynamics of auxin, TIR receptors (TIR), auxin-TIR complexes (auxinTIR), Aux/IAA mRNA (M) and protein (A), auxin-TIR-AUX/IAA complexes (auxinTIRP), ARF monomers (A) and homodimers (A2), and ARF AUX/IAA dimers (AP), according to the equations given below. Note that we take the de-dimensionalized equations, as derived in the paper. For an explanation of the meaning of parameters, values used, and units see Table S1.

Given that the model assumes constant total levels of TIR ($\alpha_{\mathrm{TIR}}$) and ARF ($\alpha_{\mathrm{ARF}}$) it follows that:


(1) $$\mathrm{auxinTIRP}=\alpha_{\mathrm{TIR}}-\mathrm{TIR}-\mathrm{auxinTIR}$$

(2) $$A_{2}=\frac{\alpha_{\mathrm{ARF}}-A-AP}{2}$$

To describe the effect of ARF monomers ($F_{1}$) and dimers ($F_{2}$) on AUX/IAA gene expression dynamics the following functions are used:


(3) $$F_{1}=\frac{A/\theta_{A}}{1+A/\theta_{A}+A_{2}/\theta_{A_{2}}+AP/\theta_{AP}+AP/\phi_{AP}+A^{2}/\phi_{A}}$$

(4) $$F_{2}=\frac{A_{2}/\theta_{A_{2}}+A^{2}/\phi_{A}}{1+A/\theta_{A}+A_{2}/\theta_{A_{2}}+AP/\theta_{AP}+AP/\phi_{AP}+A^{2}/\phi_{A}}$$

For the remaining variables the following equations are used:


(5) $$\frac{dM}{dt}=\psi (\lambda F_{1}+F_{2})-M$$

(6) $$\frac{dP}{dt}=\frac{\delta }{\eta }M-\frac{\lambda_{a}}{\eta }P\;\mathrm{auxinTIR}+\frac{\lambda_{d}}{\eta }\;\mathrm{auxinTIRP}+n(\;p_{d}AP-p_{a}AP)$$

(7) $$\frac{d\mathrm{TIR}}{dt}=\frac{-k_{a}}{\eta }\mathrm{TIRauxin}+\frac{k_{d}}{\eta }\mathrm{auxinTIR}$$

(8) $$\frac{d\mathrm{auxinTIR}}{dt}=\frac{k_{a}}{\eta }\mathrm{TIRauxin}-\frac{k_{d}}{\eta }\mathrm{auxinTIR}+\frac{\lambda_{d}+1}{\eta }\mathrm{auxinTIRP}-\frac{\lambda_{a}}{\eta }\mathrm{auxinTIR}P$$

(9) $$\frac{dA}{dt}=-2q_{a}A^{2}+2q_{d}A_{2}-p_{a}AP+p_{d}AP$$

(10) $$\frac{dAP}{dt}=p_{a}AP-p_{d}AP$$

(11) $$\frac{d\mathrm{auxin}}{dt}=\mu_{\mathrm{auxin}}(\alpha_{\mathrm{auxin}}-\mathrm{auxin})-\frac{n_{2}}{\eta }(k_{a}\mathrm{auxinTIR}-k_{d}\mathrm{auxinTIR})$$

#### Simplified Middleton 2010 model

Conservation equation:


(12) $$AP=\alpha_{ARF}-A-2A_{2}$$

Note that I used the same conservation equation for total amount of ARF being constant, but instead of using it to write an algebraic expression for ARF homodimer levels I now use it to write an algebraic expression for ARF-AUX/IAA heterodimer levels. The reason for this different choice is that I wanted to have a differential equation of ARF homodimer levels and use their dynamics as a readout for auxin signaling levels in the models.

Helper functions (same as for full model):


(13) $$F_{1}=\frac{A/\theta_{A}}{1+A/\theta_{A}+A_{2}/\theta_{A_{2}}+AP/\theta_{AP}+AP/\phi_{AP}+A^{2}/\phi_{A}}$$

(14) $$F_{2}=\frac{A_{2}/\theta_{A_{2}}+A^{2}/\phi_{A}}{1+A/\theta_{A}+A_{2}/\theta_{A_{2}}+AP/\theta_{AP}+AP/\phi_{AP}+A^{2}/\phi_{A}}$$

Differential equations:

To simplify the model and reduce the number of variables I made a number of assumptions. First, I assumed that changes in auxin level due to binding to TIR/AFB and subsequently AUX/IAA are small relative to changes from eg auxin transport and turnover and can thus be ignored. This enabled me to change auxin from a variable to a control parameter.

Next, I assumed that binding of auxin to TIR and of auxin-TIR complex to AUX/IAA protein is fast, enabling me to apply quasi-steady state approximations for equations 7 and 8 of the full model.

Solving $\frac{d\mathrm{TIR}}{dt}=0$ allows us to write:


(15) $$auxinTIR=\frac{k_{a}}{k_{d}}TIR\cdot auxin$$

Solving $\frac{d\mathrm{auxinTIR}}{dt}=0$ allows us to write


(16) $$auxinTIRP=\frac{k_{a}}{k_{d}}\frac{\lambda_{a}}{\lambda_{d}+1}P\cdot TIR\cdot auxin$$

Substituting 15 into conservation equation 1 it follows that:


(17) $$\mathrm{auxinTIRP}=\alpha_{\mathrm{TIR}}-\mathrm{TIR}-\frac{k_{a}}{k_{d}}\mathrm{TIR}\;\cdot \mathrm{auxin}$$

After reordering this gives us:


(18) $$TIR=\frac{\alpha_{\mathrm{TIR}}-auxinTIRP}{1+\frac{k_{a}}{k_{d}}auxin}$$

Substituting 18 back into 16 allows us to write:


(19) $$auxinTIRP=\frac{k_{a}}{k_{d}}\frac{\lambda_{a}}{\lambda_{d}+1}\frac{auxinP\alpha_{\mathrm{TIR}}}{2+\frac{k_{a}}{k_{d}}auxin}$$

Finally, from equation 11 of the full model, it follows that the amount of AUX/IAA protein degraded through auxin-TIR binding is the $\frac{1}{\eta }$ part of the $\frac{\lambda_{d}+1}{\eta }$ term, as it is the fraction of auxin-TIR-P complex that dissociates but does not return into the P equation.

Thus, decay of the AUX/IAA protein should be proportionate to the term I derived in equation 19. Finally, to prevent AUX/IAA protein levels going to infinity in absence of auxin I added an auxin-independent baseline decay term.

Together these simplifications result in the following 4 variables models:


(20) $$\frac{dM}{dt}=p_{b}+p_{m}(\lambda F_{1}+F_{2})-d_{m}M$$

(21) $$\frac{dP}{dt}=pM-d_{b}P-d_{auxin}\frac{auxinP\alpha_{\mathrm{TIR}}}{2+\frac{k_{a}}{k_{d}}auxin}++p_{d}AP-p_{a}A\cdot P$$

(22) $$\frac{dA}{dt}=-2q_{a}A^{2}+2q_{d}A_{2}-p_{a}A\cdot P+p_{d}AP$$

(23) $$\frac{dA_{2}}{dt}=2q_{a}A^{2}-2q_{d}A_{2}$$

I find that for these equations, using the parameter settings that in the full model result in oscillations, no oscillations occurred. A bifurcation analysis along major parameters further confirmed that this simplified model could not support oscillations. I reasoned that by performing quasi steady state assumptions and making auxin TIR and AUX/IAA protein binding instantaneous some of the delays essential for oscillations were lost (Novak and Tyson 2008). Next, I reasoned that in the current model, while auxin mediated degradation of AUX/IAA protein saturates with auxin also a saturation with protein level should occur as at some point TIR receptor levels become limiting. I therefore replaced equation 21 with the following equation:


(21*b*) $$\frac{dP}{dt}=pM-d_{b}P-d_{auxin}\frac{\alpha_{\mathrm{TIR}}auxinP}{2+\frac{k_{a}}{k_{d}}auxin+auxinP}++p_{d}AP-p_{a}A\cdot P.$$

Which enabled oscillations in our simplified model for conditions similar to those in the full model. Note that a similar approach was followed in the Mellor et al. (2016) model (see below). The meaning and value of parameters can be found in Table S2, for both oscillation supporting and non-oscillation supporting parameter settings used in the simulations.

#### Mellor 2016 model

I used the dedimensionalized version of the Mellor 2016 model. Note that the Mellor 2016 model does not consider ARF dimerization. In addition to the variables of the previous model, this model describes the dynamics of GH3 mRNA and protein (GH3m and GH3), LAX3 mRNA and protein (LAX3m and LAX3), mRNA and protein of an intermediate gene X (Xm and X), and of the auxin GH3 complex that forms prior to GH3 mediated auxin degradation (GH3aux).

Assuming a constant total level of ARF, in absence of ARF dimers the conservation equation in this model is:


(24) $$AP=\alpha_{ARF}-A$$

Helper functions:


(25) $$F_{G}=\frac{A/\theta_{A}^{ng}}{1+A/\theta_{A}^{ng}+AP/\theta_{AP}^{ng}}$$

(26) $$F_{I}=\frac{A/\theta_{A}^{ni}}{1+A/\theta_{A}^{ni}+AP/\theta_{AP}^{ni}}$$

(27) $$F_{X}=\frac{A/\theta_{A}^{nx}}{1+A/\theta_{A}^{nx}+AP/\theta_{AP}^{nx}}$$

(28) $$F_{L}=\frac{X/\theta_{X}^{nl}}{1+X/\theta_{X}^{nl}}$$

Differential equations:


(29) $$\frac{dGH3_{m}}{dt}=\mu_{G}(F_{G}-GH3_{m})$$

(30) $$\frac{dGH3}{dt}=n_{X}(GH3_{m}-GH3)-g_{a}\cdot auxin\cdot GH3+(g_{d}+g_{m})GH3auxin.$$

(31) $$\frac{dGH3auxin}{dt}=\gamma \cdot auxin\cdot GH3-(g_{d}+g_{m})GH3auxin$$

(32) $$\frac{dX_{m}}{dt}=\mu_{X}(F_{X}-X_{m})$$

(33) $$\frac{dX}{dt}=n_{X}(X_{m}-X)$$

(34) $$\frac{dM}{dt}=\mu_{I}(F_{L}-M)$$

(35) $$\frac{dP}{dt}=n_{I}M-d\frac{auxin\cdot P}{\alpha +\beta auxin+auxin\cdot P}$$

(36) $$\frac{dLAX_{m}}{dt}=\mu_{L}(F_{L}-LAX_{m})$$

(37) $$\frac{dLAX}{dt}=n_{L}(LAX_{m}-LAX)$$

(38) $$\frac{dauxin}{dt}=g_{a}\left(extauxin(1+\alpha_{LAX}LAX)-\mu_{a}auxin-GH3auxin+\frac{g_{d}}{\gamma }GH3auxin\right)$$

To study the effect of PIN mediated efflux the following equation was added:


(39) $$PIN_{efflux}=\alpha_{PIN}PIN$$

Where PIN level is either assumed constant or proportionate to X, changing equation 38 to:


(38b) $$\frac{dauxin}{dt}=g_{a}\left(\begin{array}{r} extauxin\;(1+\alpha_{LAX}LAX)-PIN_{efflux}\\ -\mu_{a}auxin-GH3auxin+\frac{g_{d}}{\gamma }GH3auxin \end{array}\right)$$

The meaning and value of parameter values can be found in Table S3

### Dynamic 1D model

#### Layout and zonation

To appropriately describe protoxylem/pericycle dynamics withing a growing root tip I developed a 1D grid-based tissue model consisting of a strand of cells. Each cell consists of a rectangle of grid points with constant width, and a height that can change due to cytoplasmic growth, cell division and vacuolar expansion. Cells are positioned longitudinally and I superimpose at distinct positions the end of the proximal meristem (where cell division occurs), and the end of the transition zone or start of the elongation zone where slow cytoplasmic growth switches to rapid vacuolar expansion driven growth (Table S4).

#### Growth division and elongation

Cell growth and expansion is modeled as in Mähönen et al. (2014). Briefly, if a cell undergoes either cytoplasmic growth or vacuaolar expansion a row of grid points is added to the shootward part of the cell, and all shootward localized cels are shifted on row upward. Cell division occurs if cells have reached double their initial size.

Unless indicated differently, cell divisions are assumed to occur perfectly symmetrically, with daughter cells inheriting exactly one half of the mother cell's auxin signaling factors. For a subset of simulations, we instead allow for a maximum 20% variation in the level of auxin signaling factors assigned to the 2 daughter cells.

#### Growth parametrization

I parametrized initial cell height, cytoplasmic growth rates and hence cell size doubling and division time, proximal meristem size, and the boundary between the transition and elongation zone such that I could approximate the root growth dynamics described in Goh et al. (2023) (Table S4). I simulated a total of 2654 μm of tissue, encompassing meristem, elongation zone, and a substantial part of the early differentiation zone.

Each cell has a differentiation variable, described by a differential equation. Initially the value of this variable is zero, and it only starts increasing once cells enter the elongation zone. Parameters of the differentiation variable are tuned such that the differentiation variable exceeds a threshold level of 85 at around 7 h, beyond which expansion ceases and cells no longer grow:


(40) $$\frac{dDiff}{dt}=p_{diff}-d_{diff}Diff$$

See Table S4 for parameter values.

To incorporate some stochasticity, instead of making all cells divide synchronously at a constant rate, I incorporated a bottom stem cell and initial cell, with slower division rates than the rest of the meristematic transit amplifying cells (Table S4). Additionally, each time a cell divides, daughter cells receive a division time in between 95% and 105% of the average division time for their respective position.

Given the focus on cell-autonomous rather than tissue level emergent oscillation dynamics, I did not take into account the dependence of cell division, elongation and differentiation dynamics on auxin signaling or vice versa that could potentially lead to emergent, tissue level phenomena.

#### Intracellular dynamics and parametrization

Each cell contains the auxin signaling network as described by the simplified Middleton 2010 model. All cells are parametrized identically using the values described in Table S2, except for the total amount of ARF, which is used to control the capacity of the cells to oscillate. In our simulations I use either high ARF, oscillation promoting, levels (1.5) in the meristem and transition zone or above the transition zone. Elsewhere low ARF, non-oscillation promoting levels (0.5) are used. Upon cell division, daughter cells inherit the state of their mother cell.

The current model ignores auxin transport dynamics between cells. Other studies have investigated the potential role for auxin transport and its regulation in auxin oscillations. First, a previous study incorporating the dual dependence of PIN levels on auxin, with lower auxin levels promoting PIN gene expression and higher auxin levels promoting PIN protein degradation demonstrated a so-called reflected flow mechanism that combined with temporally increasing auxin influx can lead to localized auxin oscillations (Mironova et al. 2010). Similarly, a study incorporating so-called up-the-gradient PIN polarization combined with cell division demonstrated regular auxin oscillations under sufficiently synchronized divisions (Bellows et al. 2023). In both cases, oscillations arise from tissue level interactions and are thus emergent rather than cell autonomous. Therefore, these types of interactions between auxin and auxin transport were not considered in the current model.

However, in absence of auxin transport dynamics, the standard model used in this study imposes a single constant auxin level on each cell, inconsistent with the auxin gradients experimentally observed in the Arabidopsis root. Therefore, to validate the followed approach, also a model variant incorporating PIN mediated auxin transport was created. Notably, simply incorporating downward PIN1 mediated auxin transport would be insufficient to generate an auxin gradient, and instead results in a single tip-localized auxin maximum. Expanding the auxin transport by also having lateral auxin influx to emulate a reflux-loop type topology (as previously done in eg Van den Berg et al. 2021) improves this into an auxin gradient in the root tip with a secondary rise in auxin levels in the elongation zone. The model with transport was parametrized such that while auxin levels vary spatially, they are of the same order of magnitude as in the non-auxin transporting default model (Fig. S6). For settings with high ARF levels in the meristem, as before oscillations occur in the meristematic zone but with more irregular dynamics. As a consequence, after memorization the alternating PBS non-PBS pattern is also a bit less regular, but no fundamentally different behavior occurs (Fig. S6a). For settings with high ARF levels in the elongation zone, as before oscillations only started in the elongation zone, yet showed slightly different dynamics especially higher up in the root (Fig. S6b). Still, these changes do not lead to out of phase oscillations and hence after memorization give rise to similar patterns with either all cells forming a PBS (shown) or, when timing memorization differently, no cells do so. Of course, the auxin transport could be parametrized such as overriding the superimposed ARF pattern's control on where oscillations occur, just like one could implement an auxin level rather than ARF pattern to control oscillation location in the standard model. Thus, auxin transport does not fundamentally affect model outcomes and for simplicity in the manuscript the standard model without auxin transport was used.

#### Synchronization of oscillations

In order for individual cells in a tissue to synchronously oscillate despite eg molecular noise, cell-cell communication is critical. However, while for the somitogenesis field delta-notch signaling has been identified as the molecular machinery underlying this synchronization (Horikawa et al. 2006), for the hypothetical auxin oscillator no candidate communication mechanism has been proposed. Cell-cell coupling could arise from either transport mediated auxin exchange but also exchange of proteins or mRNA through plasmodesmata could play a role. Additionally, depending on the delay and strength of cell-cell signaling, tissue level oscillations may become either faster or slower than cell-autonomous oscillations (Herrgen et al. 2010). Because of the unclarity on molecular machinery underlying cell-cell communication as well as the additional complexities this introduces, I refrained from explicitly modeling synchronizing cell-cell communication. Instead, at the start of simulations all cells in the tissue are simply started from identical conditions, implicitly modeling their synchronization through communication. Combined with the inheritance of the mothers’ state by daughter cells this ensures tissue level synchronization.

#### Special settings

Superimposed mRNA oscillations (Fig. 9):

For these simulations we replaced equation 21 with:


(20*b*) $$\frac{dM}{dt}=p_{o}(sin(\omega t)-d_{m}M$$

With $p_{o}=0.125p_{m}$ to compensate for the fact that a sine function has a maximum of 1 while $\lambda F_{1}+F_{2}$ typically stays below 0.35, and . From ω=2πf. it then follows that f=0.25cycles/h resulting in a period of 4 hours.

Periodic stimuli (Fig. 10):

For these simulations we replace the constant value of auxin with the equation:


(41) auxin=4+3sin(ωt)

where the values 4 and 3 were chosen to have auxin input vary between 1 and 7, and using for *ω* either a value of 1.50 or 1.05 *radians/h*, and hence f a value of 0.238 or 0.167 *cycles/h*, resulting in a period of 4.2 h or 6 h

Additional sources of noise:

In addition to investigating the effects of noisy division of auxin signaling factors across the 2 daughter cells upon division, also the consequences of noisy gene expression and auxin input were investigated. For gene expression, we augmented equation 20 with a noise term (Ito form of Wiener process), using the Euler-Marayama method for integration and a noise strength of 0.00015.

For cellular auxin input, after each division event a cell was assigned an auxin value drawn from a distribution with the default auxin value used in the other simulations as a mean and minimum values 20% below and maximum values 20% above this mean.

Cryptic oscillator:

To investigate whether a cryptic, non-auxin signaling based oscillator could drive EZ auxin signaling to result in EZ auxin signaling oscillations I added a simple phase oscillator to our model:


(42) $$\frac{d\varphi }{dt}=\omega$$

With ω=0.0000694rad/s and φ=φmod1to ensure phase to vary with an approximately 4 h period between 0 and 1.

From the state of the phase oscillator, I then compute a help variable:


(43) $$f=\frac{1+sin(2\pi \varphi )}{2}$$

Which converts phase into a sine wave with an approximately 4 h period and value between 0 and 1.

This help variable is subsequently used to affect auxin signaling in EZ by affecting AUX/IAA transcription. For this equation 20 in the EZ was replaced by:


(44) $$\frac{dM}{dt}=p_{b}+p_{m}(\lambda F_{1}+F_{2})-d_{m}M\;+\alpha \;f$$

With *α* the strength of the coupling between the cryptic oscillator and auxin signaling. For auxin signaling in MZ we maintain the original equation 20. To investigate the impact of coupling strength on EZ auxin signaling dynamics I varied the value of *α* between 0.00001 and 0.0001, which correspond to approximately a factor 0.1 or a factor 1 of the impact of the ARF (term $p_{m}(\lambda F_{1}+F_{2})$) on AUX/IAA transcription.

#### Finite domain size

Cells exceeding a certain threshold distance from the start of the tissue are removed from the simulation. This enables us to maintain a constant simulation domain size despite continuous growth.

#### Numerics

ODEs are solved using simple Euler forward integration with a timestep of 0.025 s. Spatial resolution of the grid was 2 micron.

### Model simulation and analysis

The single cell full Middleton 2010 model, simplified Middleton 2010 model and Mellor 2016 model were coded in python and numerically solved using the function odeint (Virtanen et al. 2020). These codes were used to generate the single cell temporal dynamics shown in Figs 4–6. Additionally, for bifurcation analysis (Fig. 4) the full and simplified Middleton models were coded in R, making use of the package custom script Grind.R (created by Rob J. de Boer, for information and download see https://bioinformatics.bio.uu.nl/rdb/grind.html), which in turn uses the package deSolve for numerically solving the equations (Soetaert et al. 2010).

The 1D model was coded in C++, using simple Euler forward integration with a timestep of 0.25 s and a space step of 1 μm. The spatiotemporal data generated by this code was plotted using separate python codes.

## Supplementary Material

koag213_Supplementary_Data

## Data Availability

All code used for the simulations described in this paper is publicly available from either: https://www-binf.bio.uu.nl/khwjtuss/UnlikelihoodClock/ or from: https://github.com/kirstentt/-un-likelyclocks.
