# Supplementary material for: The (un)likelihood of clock-driven lateral root priming; a modeling exploration
Source: Plant Cell. 2026 Jul 14;38(7):koag213. doi: 10.1093/plcell/koag213 (PMC13421895; doi:10.1093/plcell/koag213)
Supplement: koag213_Supplementary_Data [file koag213_supplementary_data.zip › SupplementaryTable1.pdf]

**Supplementary Table 1 Parameters for the original Middleton 2010 model.** Parameter values of the dedimensionalised model as derived in Middleton et al. (2010) are used, hence parameters are dimensionless. Symbols, meaning and values are provided.

| *Parameter* | *Meaning* | *Value* |
|---|---|---|
| $\alpha_{TIR}$ | Total TIR amount | 1.0 |
| $\alpha_{ARF}$ | Total ARF amount | 1.0 |
| $\theta_A$ | Affinity constant for A | 0.1 |
| $\theta_{A2}$ | Affinity constant for A2 | 0.01 |
| $\theta_{AP}$ | Affinity constant for AP | 0.1 |
| $\varphi_{AP}$ | Cooperativity constant for AP | 0.1 |
| $\varphi_A$ | Cooperativity constant for A | 0.1 |
| $\psi$ | mRNA production scaling | 100 |
| $\lambda$ | Weight of F1 in M dynamics | 0.1 |
| $\delta$ | Protein production rate | 1 |
| $\eta$ | Scaling factor for protein/complex dynamics | 0.01 |
| $\lambda_a$ | AuxinTIR P association constant | 10 |
| $\lambda_d$ | AuxinTIRP dissociation constant | 1 |
| $n$ | Stoichiometric coefficient | 1 |
| $p_a$ | AP association constant | 100 |
| $p_d$ | AP dissociation constant | 100 |
| $k_a$ | Auxin–TIR association constant | 0.1 |
| $k_d$ | Auxin–TIR dissociation constant | 1 |
| $q_a$ | ARF dimerization rate | 1 |
| $q_d$ | ARF dimer dissociation rate | 1 |
| $\mu_{auxin}$ | Auxin turnover rate | 10 |
| $\alpha_{auxin}$ | Relative auxin production constant | 1 |
| $n_2$ | Stoichiometric factor in auxin–TIR dynamics | 10 |
