# Supplementary material for: The (un)likelihood of clock-driven lateral root priming; a modeling exploration
Source: Plant Cell. 2026 Jul 14;38(7):koag213. doi: 10.1093/plcell/koag213 (PMC13421895; doi:10.1093/plcell/koag213)
Supplement: koag213_Supplementary_Data [file koag213_supplementary_data.zip › SupplementaryTable2.docx]

**Supplementary Table 2 parameters for the simplified Middleton 2010 model**

“Value-osc” are the parameter values used to simulate oscillatory dynamics. “Value-non-osc” are parameter values to generate non-oscillatory dynamics. “-“ indicates parameter values identical to the oscillatory regime are used, only distinct parameter values are provided for clarity.

| Parameter | Meaning | Value -osc | Value- non-osc |
| --- | --- | --- | --- |
| Auxin | Auxin concentration | 5.0 | - |
| $\alpha_{TIR}$ | Total TIR amount | 1.0 | - |
| $\alpha_{ARF}$ | Total ARF amount | 1.5 | 0.375 |
| $\theta_{A}$ | Affinity constant for A | 0.1 | - |
| $\theta_{A2}$ | Affinity constant for A2 | 0.01 | - |
| $\theta_{AP}$ | Affinity constant for AP | 0.1 | - |
| $\varphi_{AP}$ | Cooperativity constant for AP | 0.1 | - |
| $\varphi_{A}$ | Cooperativity constant for A | 0.1 | - |
| $p_{b}$ | Baseline mRNA production rate | 0 |  |
| $p_{m}$ | Maximum ARF mediated mRNA production rate | 10 | - |
| $\lambda$ | Weight of F1 in M dynamics | 0.1 | - |
| $d_{m}$ | mRNA degradation rate | 1 |  |
| $\delta$ | Protein production rate | 10 | 2.5 |
| $d_{b}$ | Baseline protein degradation rate | 0.1 | - |
| $d_{auxin}$ | Auxin mediated protein degradation rate | 100 | - |
| $p_{a}$ | AP association constant | 100 | - |
| $p_{d}$ | AP dissociation constant | 100 | - |
| $k_{a}$ | Auxin–TIR association constant | 0.2 | - |
| $k_{d}$ | Auxin–TIR dissociation constant | 1 | - |
| $q_{a}$ | ARF dimerization rate | 1 | 10 |
| $q_{d}$ | ARF dimer dissociation rate | 1 | 10 |
