# Supplementary material for: The (un)likelihood of clock-driven lateral root priming; a modeling exploration
Source: Plant Cell. 2026 Jul 14;38(7):koag213. doi: 10.1093/plcell/koag213 (PMC13421895; doi:10.1093/plcell/koag213)
Supplement: koag213_Supplementary_Data [file koag213_supplementary_data.zip › SupplementaryTable3.docx]

**Supplementary Table 3 Parameters for the Mellor 2016 model** Note that as for the original and simplified Middleton model, the dedimensionalized version of the model is used, resulting in dimensionless parameters. As before symbols, meaning and values are provided.

| Parameter | Meaning | Value |
| --- | --- | --- |
| $\alpha_{LAX}$ | LAX3 mediated influx rate | 72.5134 |
| $\alpha_{PIN}$ | PIN mediated efflux rate | 0.1 |
| $\mu_{aux}$ | GH3 independent auxin degradation rate | 1.9 x 10^-6^ |
| $\gamma$ | Maximum GH3 protein level | 0.40952 |
| $\mu_{g}$ | GH3 mRNA production rate | 0.0236 |
| $n_{g}$ | GH3 protein production rate | 0.0137 |
| $g_{a}$ | Auxin GH3 association rate | 5987.2 |
| $g_{d}$ | Auxin-GH3 complex dissociation rate | 128.228 |
| $g_{m}$ | GH3 mediated auxin degradation | 21422 |
| $\mu_{i}$ | AUX/IAA mRNA production rate | 0.2497 |
| $\delta_{i}$ | AUX/IAA protein production rate |  |
| $n_{i}$ | Auxin mediated AUX/IAA protein degradation rate | 176.4252 |
| $\mu_{x}$ | X mRNA production rate | 12.9646 |
| $n_{x}$ | X protein production rate | 0.2384 |
| $\mu_{l}$ | LAX mRNA production rates | 0.5472 |
| $n_{l}$ | LAX protein production rate | 5.1818 |
| $\theta_{ia}$ | Affinity constant of IAA promotor for ARF | 0.2356 |
| $\theta_{ir}$ | Affinity constant of IAA promotor for ARF-IAA | 3.0792 |
| $\theta_{xa}$ | Affinity constant of X promotor for ARF | 0.1615 |
| $\theta_{xr}$ | Affinity constant of X promotor for ARF-IAA | 0.2024 |
| $\theta_{la}$ | Affinity constant of LAX promotor for X | 1.7043 |
| $\theta_{ga}$ | Affinity constant of GH3 promotor for ARF | 0.0494 |
| $\theta_{gr}$ | Affinity constant of GH3 promotor for ARF-IAA | 0.0181 |
| $\alpha$ | Constant modulating auxin driven IAA degradation | 238580 |
| $\beta$ | Constant modulating auxin driven IAA degradation | 0.6467 |
