# Supplementary material for: The (un)likelihood of clock-driven lateral root priming; a modeling exploration
Source: Plant Cell. 2026 Jul 14;38(7):koag213. doi: 10.1093/plcell/koag213 (PMC13421895; doi:10.1093/plcell/koag213)
Supplement: koag213_Supplementary_Data [file koag213_supplementary_data.zip › SupplementaryTable4.pdf]

Supplementary Table 4 Parameters for the growing1D model

Parameter	*Meaning*	*Value*	*Units*
B_{MZ-TZ}	Boundary between proximal meristem and transition zone	155	microm
B_{TZ-EZ}	Boundary between transition zone and elongation zone	200	microm
H_{init}	Initial cell height	4	microm
H_{div}	Cell height at which division takes place	9	microm
H_{max}	Maximum cell height used to compute elongation rate	100	microm
t_{div}	Time it takes for transit amplifying cell to grow from H_{init} to H_{div} and undergo division	11	h
$t_{,stem}$	Division time of stem cell	30	h
$t_{,initial}$	Division time of initial cell	17	h
t_{elong}	Time it takes for a cell to grow from $2H_{init}$ to H_{max}	18	h
T_{diff}	Differentiation threshold beyond which cells no longer elongate	85	a.u.
p_{diff}	Rate of increase of differentiation variable	0/0.0024	s-1
d_{diff}	Turnover rate of differentiation variable	0.000024	s-1
