# Supplementary material for: The (un)likelihood of clock-driven lateral root priming; a modeling exploration
Source: Plant Cell. 2026 Jul 14;38(7):koag213. doi: 10.1093/plcell/koag213 (PMC13421895; doi:10.1093/plcell/koag213)
Supplement: koag213_Supplementary_Data [file koag213_supplementary_data.zip › SupplementaryTable5.pdf]

Supplementary Table 5 Effect of parameters on oscillation characteristics. Shown are the parameters of the simplified Middleton model developed in this study that were varied to obtain oscillation amplitude differences. Note that θ_A is left out from the table because of its limited effect on oscillation amplitude.

parameter	Low/High amplitude values	Mean auxin signalling	Nr of cycles to 95% of high amplitude	Immediate/ Long term amplitude increase	Low/High amplitude period (h)
Auxin	3-10	0.026-0.032	4-5	0.048-0.065 (1.35-fold) 0.048-0.12 (2.5-fold)	2.9-4.8
α_{TIR}	0.4-1	0.011-0.03	3-4 overshoot	0.03-0.10 (3.3 3fold) 0.03-0.09 (3-fold)	4.9 - 4.1
α_{ARF}	1-1.5	0.017-0.029	9-10	0.029-0.039 (1.34-fold) 0.029-0.093 (3.21-fold)	2.6-4.1
θ_{A2}	0.0135-0.01	0.032-0.029	11-12 First 4 smaller than low amplitude one	0.045-0.033 (0.73-fold) 0.045-0.093 (2.07-fold)	2.5-4.1
θ_{AP}	0.047-0.1	0.037-0.029	9-10 First 2 smaller than low amplitude one	0.055-0.044 (0.8-fold) 0.055-0.093 (1.69-fold)	2.5-4.1
φ_{AP}	0.05-0.1	0.036-0.029	8-9	0.061-0.060 (0.98-fold) 0.061-0.093 (1.52-fold)	2.6-4.1
φ_A	0.05-0.1	0.024-0.029	7-8	0.041-0.049 (1.20-fold) 0.041-0.093 (2.27-fold)	2.7-4.1
p_b	0.48-0	0.013-0.029 (2.23-fold increase)	7-8	0.018-0.049 (2.72-fold) 0.018-0.095 (5.3-fold)	3.7-4.1
p_m	75-10	0.004-0.029 (7.25-fold increase)	3-4 overshoot	0.02-0.13 (6.5-fold) 0.02-0.095 (4.75-fold)	6.5-4.1
λ	0.375-0.05	0.023-0.030	8-9	0.042-0.053 (1.26-fold)	2.9-4.2

				0.042-0.099 (2.36-fold)	
d_m	1.35-0.5	0.034-0.017	9-10	0.048-0.039 (0.81-fold) 0.048-0.11 (2.29-fold)	2.3-8
δ	68-100	0.038-0.029	8-9	0.052-0.049 (0.94-fold) 0.52-0.093 (1.79-fold)	2.5-4.1
d_b	0.43-0.1	0.026-0.029	9-10	0.035-0.038 (1.09-fold) 0.035-0.093 (2.66-fold)	2.6-4.1
d_{auxin}	40-100	0.011-0.030 (2.73-fold increase)	3-4 overshoot	0.027-0.10 (3.7-fold) 0.027-0.10 (3.7-fold)	4.9-4.1
