# Supplementary material for: The (un)likelihood of clock-driven lateral root priming; a modeling exploration
Source: Plant Cell. 2026 Jul 14;38(7):koag213. doi: 10.1093/plcell/koag213 (PMC13421895; doi:10.1093/plcell/koag213)
Supplement: koag213_Supplementary_Data [file koag213_supplementary_data.zip › Supplementary_legends.docx]

**Supplementary Figure Legends**

**Supplementary Figure 1 Explanation of Bifurcation diagrams.** A bifurcation diagram (central diagram) plots how the number and stability of the equilibria of a model change as a function of changing the value of a single model parameter. Thus, for a single model with multiple parameters, multiple bifurcation diagrams can be created. The value of the parameter that is varied is displayed on the x-axis. On the y-axis one plots for one of the variables of the model its equilibrium value and if relevant the amplitude of its oscillations. In this example, our model contains two variables, mRNA and protein, and we plot mRNA values on the y-axis of the bifurcation plot as a function of increasing the parameter controlling the delay of protein repressing mRNA production on the x-axis. For the parameter value shown under 1, the system converges to a stable equilibrium (red dot) and that equilibrium value is plotted. As the delay parameter increases in 2, the system converges to a higher level stable equilibrium (red dot) and we plot this equilibrium value. By continuously varying the parameter value between the values under 1 and 2 we obtain the red line in the bifurcation diagram. In 3, upon increasing the parameter value further the equilibrium becomes unstable (blue dot), and in addition to plotting this equilibrium value we now also plot the minimum and maximum value of the stable oscillations. Finally, in 4, upon increasing the delay even further, the location of the stable equilibrium has not shifted much (blue dot), yet oscillation amplitude is increased. By continuously varying the parameter value between the values shown for 3 and 4 we obtain both the blue and black lines in the bifurcation diagram.

**Supplementary Figure 2 EZ localised oscillations in presence of noise do not generate patterned PBS.** An ARF gradient supporting auxin signalling oscillations in the EZ was imposed, and 3 different sources of noise were added to investigate if this results in sufficient phase differences to pattern alternating PBS and non-PBS (high and low memorized auxin levels). 20% cell division noise (A, B, C), 10% gene expression noise (D, E, F), and 20% auxin input noise (G, H, I) were implemented (For details see methods), and spatiotemporal auxin signalling dynamics (A, D, G), spatiotemporal auxin signalling dynamics incorporating memorisation in the elongation zone (B, E, H), and auxin signalling dynamics as a function of distance from the tip for 4 different consecutive cells (C, F, I) were plotted (line of fourth cell coincides with one of the other cells). In all three cases limited phase differences arose, even after several oscillation cycles, and memorisation resulted in minimal differences in final values. Gene expression noise reduced oscillation amplitude.

**Supplementary Figure 3 Clockwork versus stopwatch like oscillatory behavior.** A) If oscillations occur in the stem cell niche and meristem, through inheritance of oscillator phase after division (see growing and dividing clocks) a global time keeping arises (look at clock state in bottom row from left to right), and cells arriving at different times do so with different clock phases. B) If oscillations start in the elongation zone, all cells in the stem cell niche and meristem are in a steady state, like a stopwatch not yet pressed to start, and all cells start with the same clock state when entering the elongation zone, like when crossing the start and a stopwatch is pressed to run.

**Supplementary Figure 4 MZ localised oscillations in presence of noise maintain phase memory.** An ARF gradient supporting auxin signalling oscillations in the MZ was imposed, and 3 different sources of noise were added to investigate if this perturbs global phase memory and alternating PBS and non-PBS patterning. 10% cell division noise (A, B), 10% gene expression noise (C, D), and 20% auxin input noise (E, F) were implemented (For details see methods), and spatiotemporal auxin signalling dynamics (A, C), and spatiotemporal auxin signalling dynamics incorporating memorisation in the elongation zone (B, D) were plotted. In all three cases MZ oscillation dynamics and memorisation patterns were affected, yet overall characteristics were maintained. Cell division noise has strongest effect on MZ oscillations. Gene expression noise reduced oscillation amplitude.

**Supplementary Figure 5 Cryptic oscillations require strong coupling to drive auxin signalling oscillations** Paired spatiotemporal auxin signalling dynamics and auxin signalling dynamics as function of distance from the tip for 4 consecutive cells (line of fourth cell coincides with one of the other cells) are shown. On the left dynamics are shown for the cryptic oscillator feeding into auxin signalling dynamics that autonomously is not oscillating (lower ARF value), On the right dynamics are shown if auxin signalling dynamics in isolation also generates oscillatory dynamics (higher ARF value). From bottom to top coupling strength was varied from 0.1, 0.3, 0.5 to 1.0 the value of average ARF mediated input to AUX/IAA expression (for details see Methods). While for all 4 coupling strengths and both auxin signalling settings out of phase EZ oscillations result, substantial amplitudes were only obtained for strong coupling. Still amplitude was significantly less than obtained for MZ originating auxin signalling oscillations.

**Supplementary Figure 6 Model dynamics are robust against PIN mediated auxin patterning.** An ARF gradient supporting MZ (A, B, C) or an ARF gradient supporting EZ oscillations (D, E, F) was implemented as before. Instead of a single constant auxin level for all cells (5, in arbitrary units), auxin levels now depended on PIN mediated auxin transport and a simplified reflux loop (see methods). Shown are the resulting auxin gradients, which are identical as they are not affected by ARF levels (A, D), the spatiotemporal auxin patterns (B, E) and the spatiotemporal auxin patterns including memorisation in the elongation zone (C, F). Resulting oscillatory auxin signalling patterns are more irregular, yet after memorization do not result in fundamentally different patterns as previously obtained for a homogeneous constant auxin level.

**Supplementary Table legends**

**Supplementary Table 1 Parameters for the original Middleton 2010 model.** Parameter values of the dedimensionalised model as derived in Middleton et al. (2010) are used, hence parameters are dimensionless. Symbols, meaning and values are provided.

**Supplementary Table 2 parameters for the simplified Middleton 2010 model**

“Value-osc” are the parameter values used to simulate oscillatory dynamics. “Value-non-osc” are parameter values to generate non-oscillatory dynamics. “-“ indicates parameter values identical to the oscillatory regime are used, only distinct parameter values are provided for clarity.

**Supplementary Table 3 Parameters for the Mellor 2016 model** Note that as for the original and simplified Middleton model, the dedimensionalized version of the model is used, resulting in dimensionless parameters. As before symbols, meaning and values are provided.

**Supplementary Table 4 Parameters for the growing1D model**

**Supplementary Table 5 Effect of parameters on oscillation characteristics**.

Shown are the parameters of the simplified Middleton model developed in this study that were varied to obtain oscillation amplitude differences. Note that $\theta_{A}$ is left out from the table because of its limited effect on oscillation amplitude.
