# Supplementary material for: The (un)likelihood of clock-driven lateral root priming; a modeling exploration
Source: Plant Cell. 2026 Jul 14;38(7):koag213. doi: 10.1093/plcell/koag213 (PMC13421895; doi:10.1093/plcell/koag213)
Supplement: koag213_Supplementary_Data [file koag213_supplementary_data.zip › SupplFig5_new.pdf]

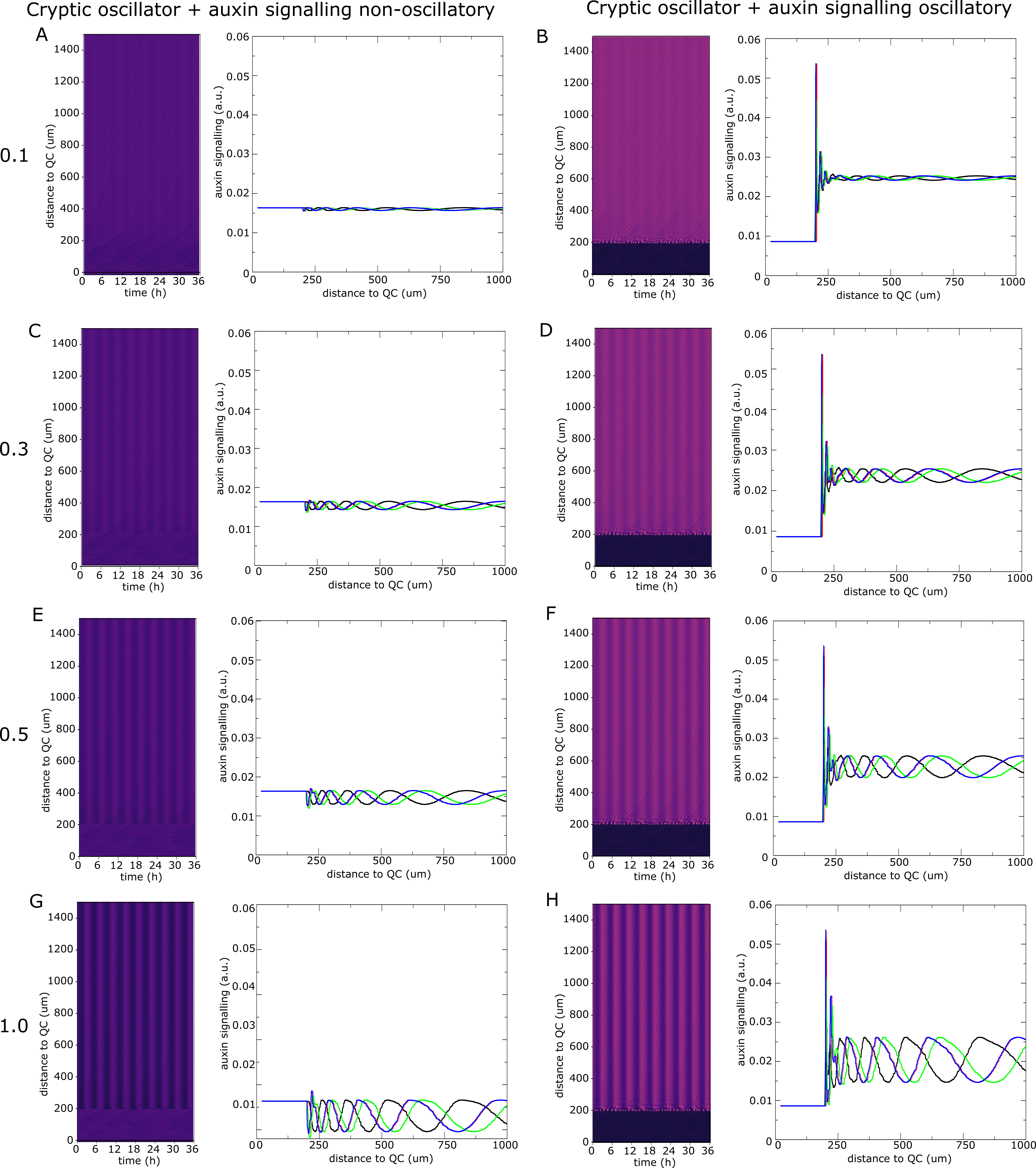
Cryptic oscillator + auxin signalling non-oscillatory
Cryptic oscillator + auxin signalling oscillatory
A
B
C
D
E
F
G
H
0.1
0.3
0.5
1.0
distance to QC (um)
time (h)
auxin signalling (a.u.)
distance to QC (um)
