# Supplementary figures and images for: The (un)likelihood of clock-driven lateral root priming; a modeling exploration
Source: Plant Cell. 2026 Jul 14;38(7):koag213. doi: 10.1093/plcell/koag213 (PMC13421895; doi:10.1093/plcell/koag213)

①

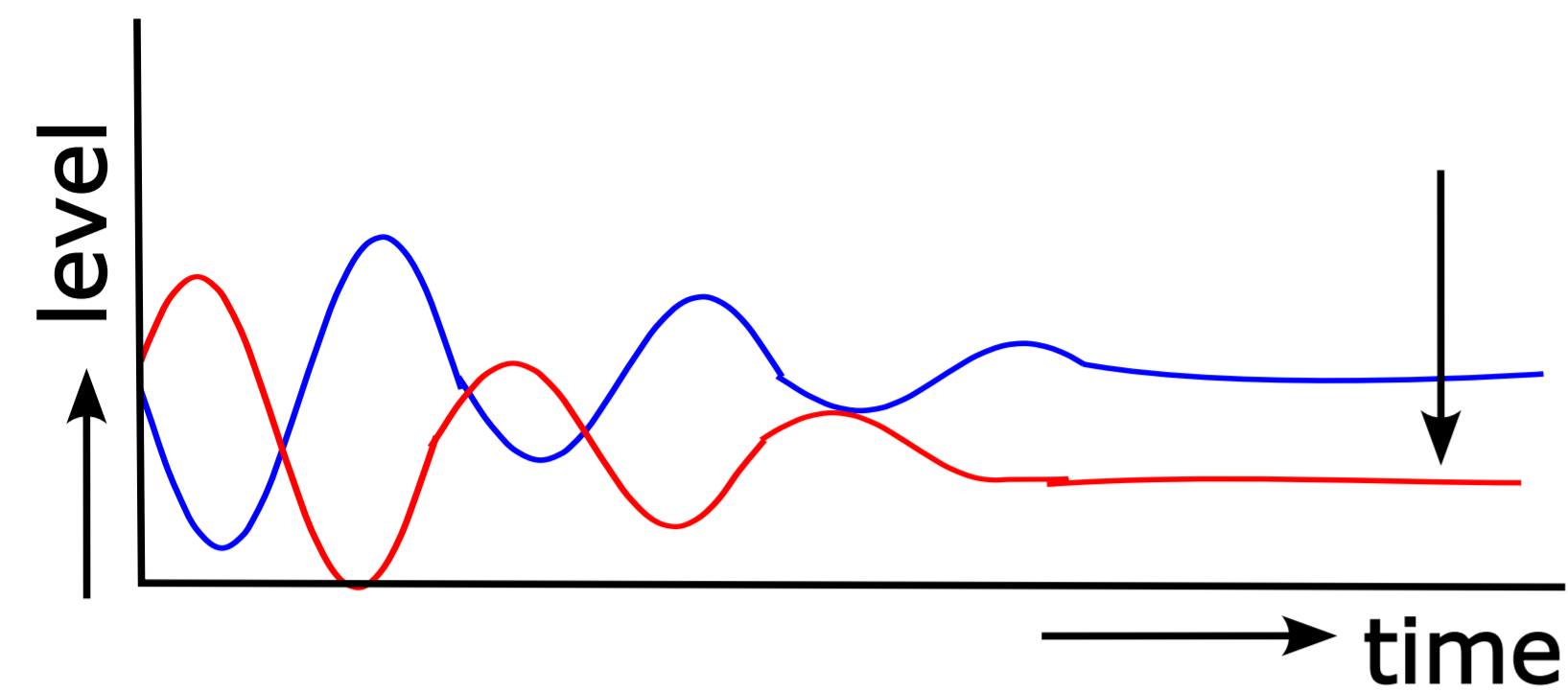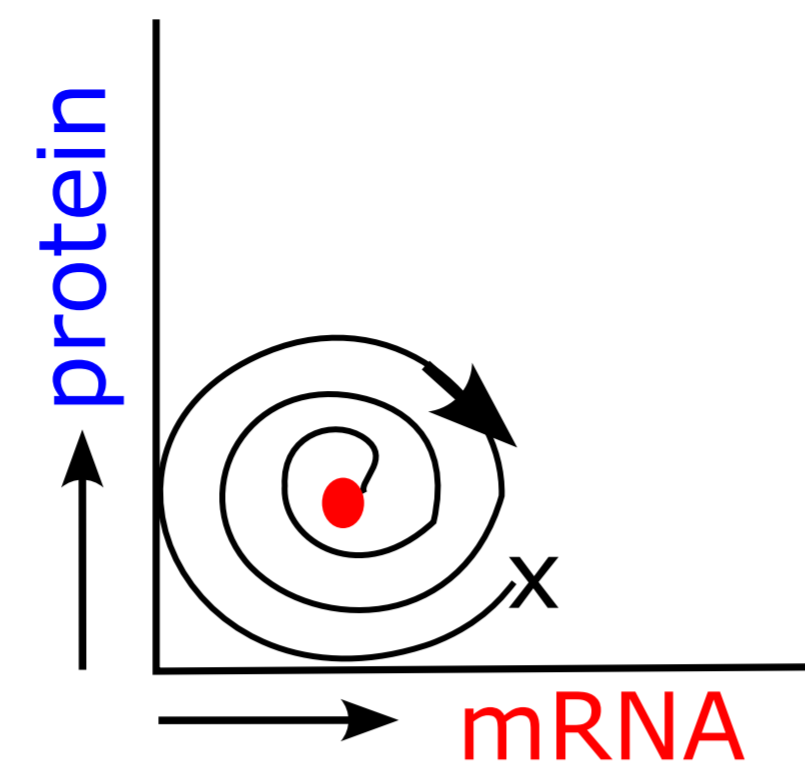

②

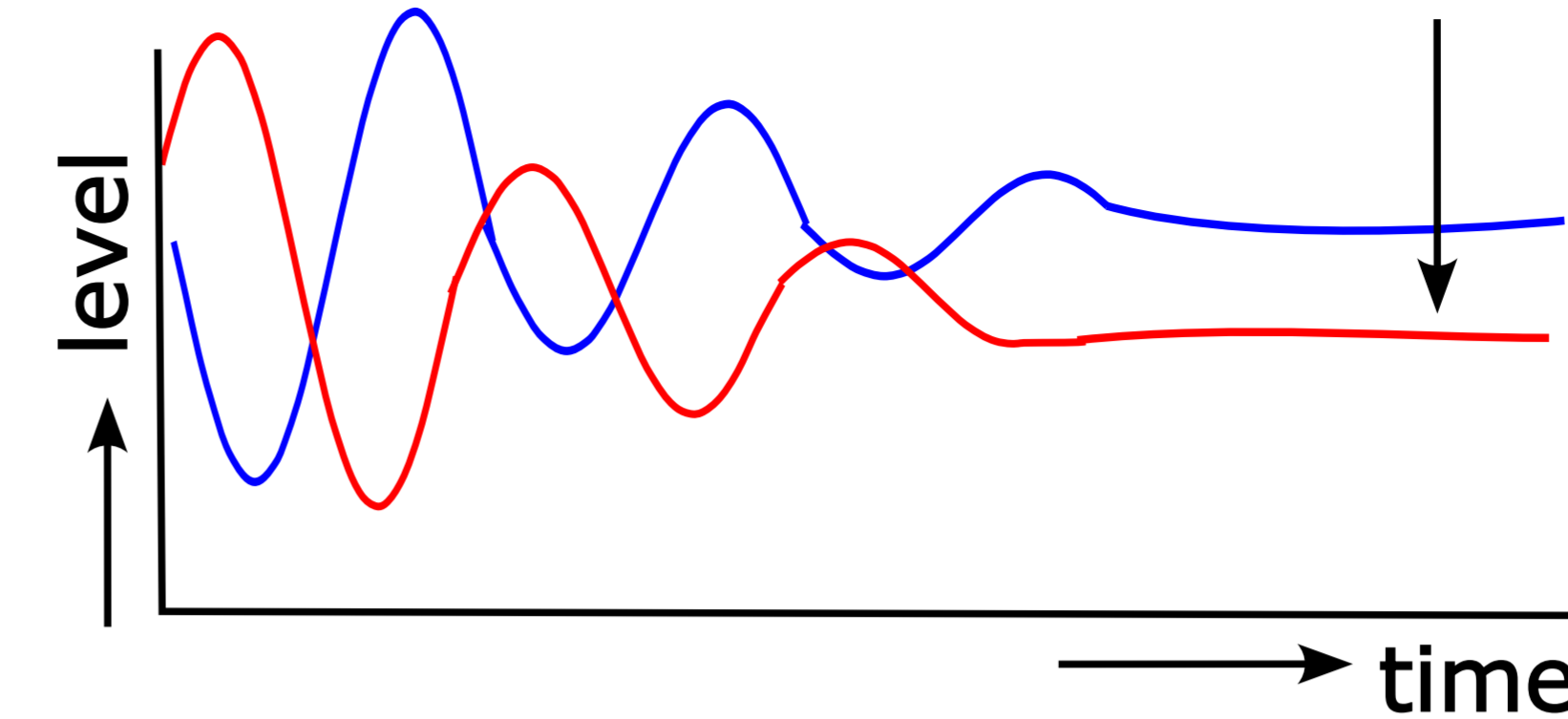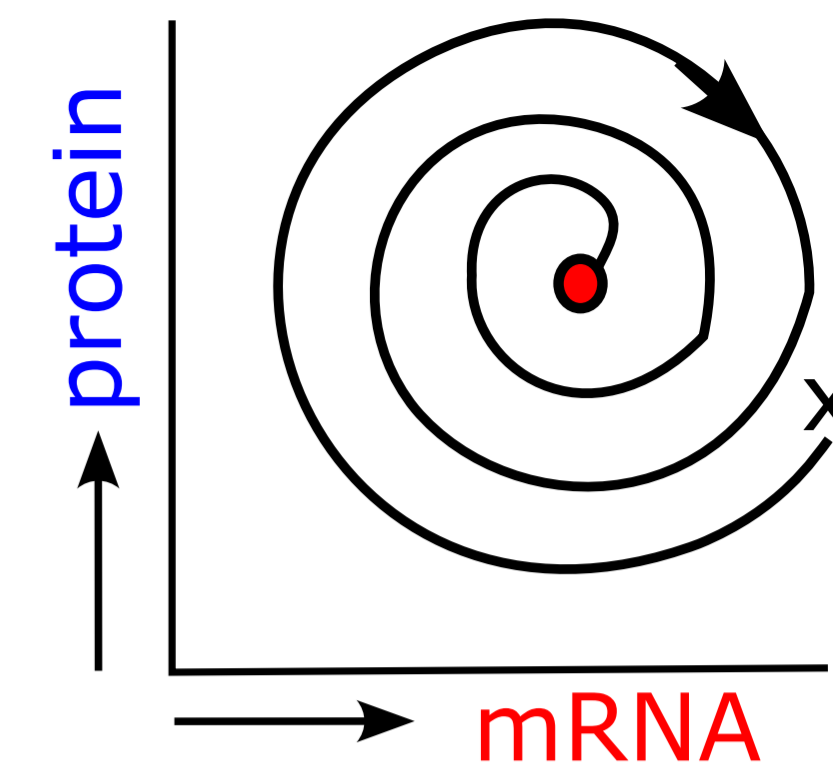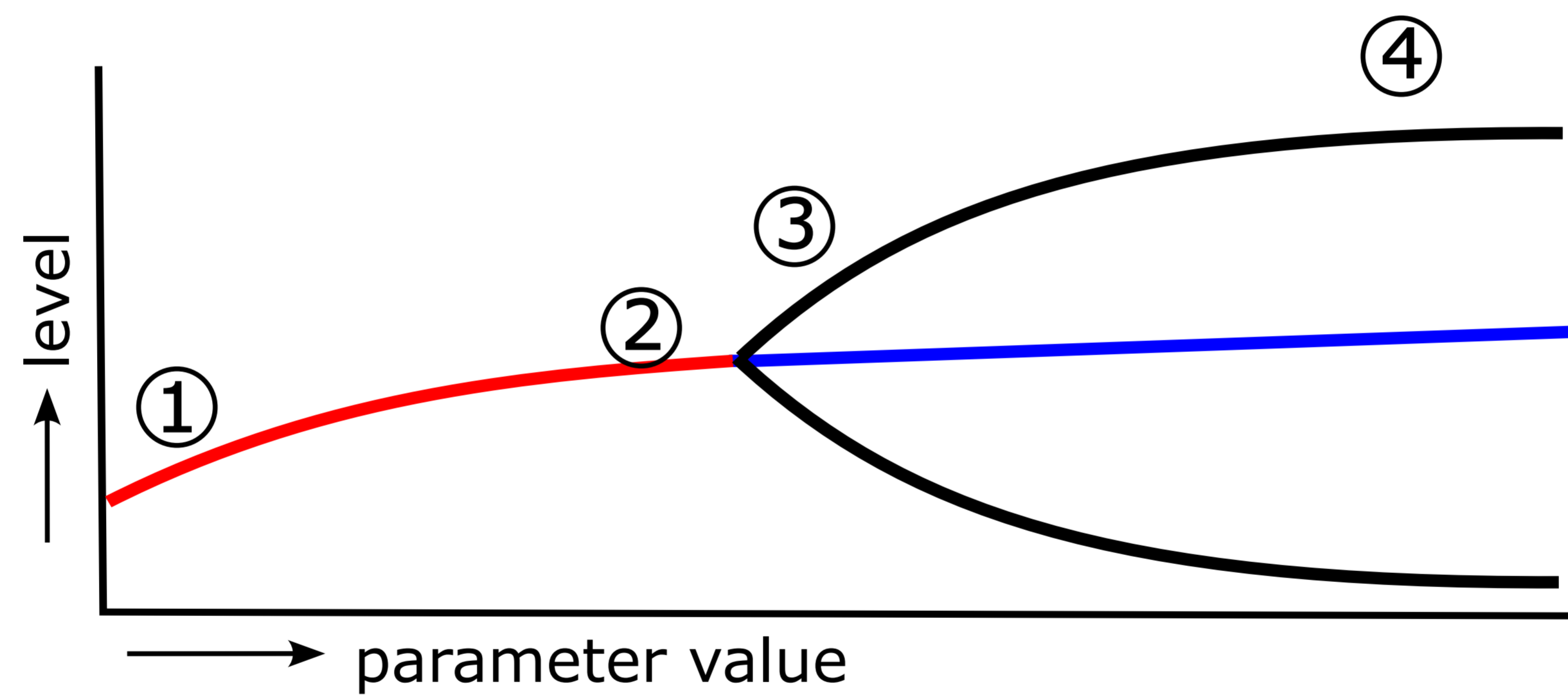

- stable steady state
- unstable steady state
- min/max oscillations

③

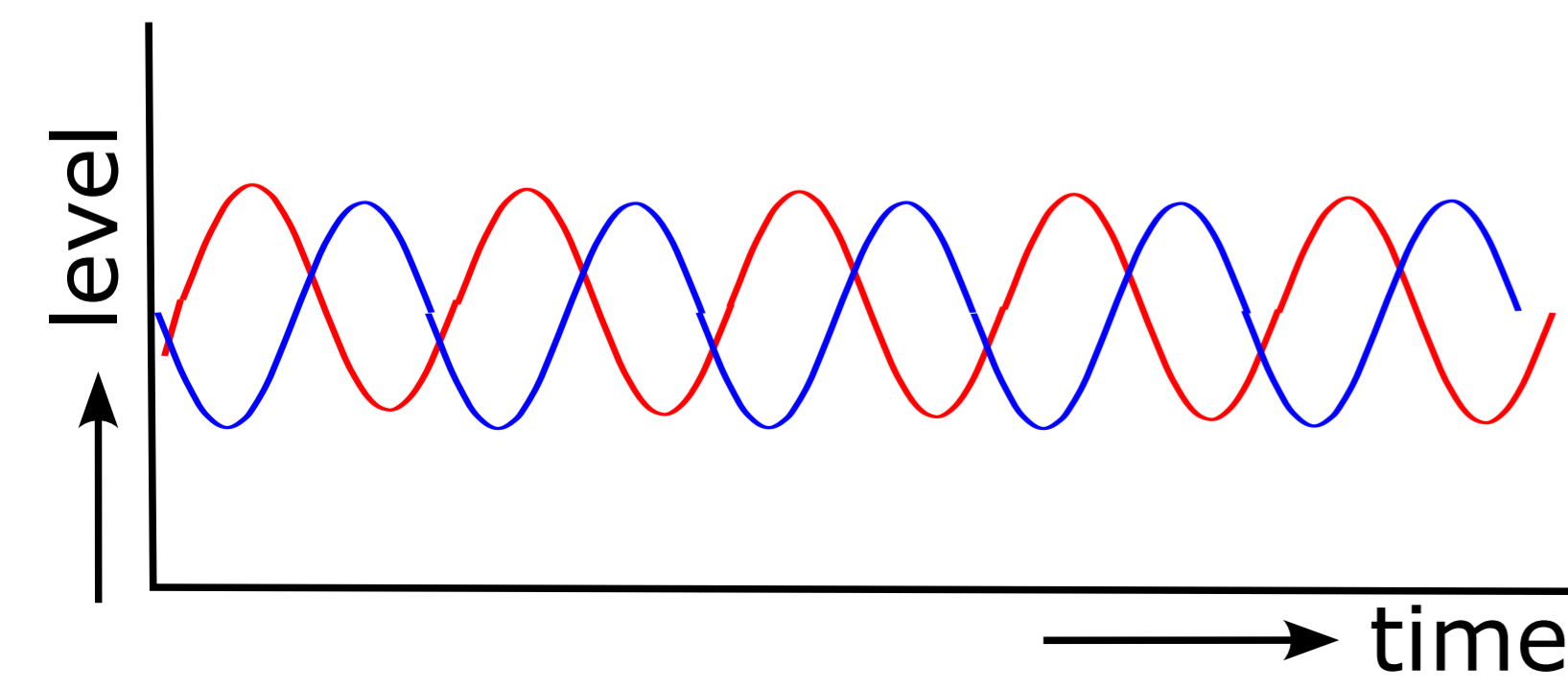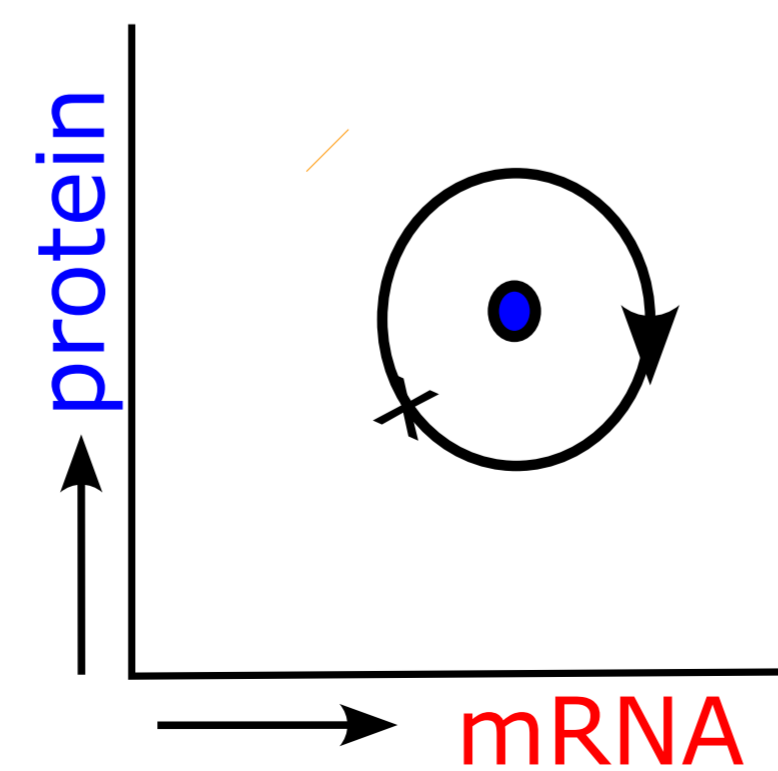

④

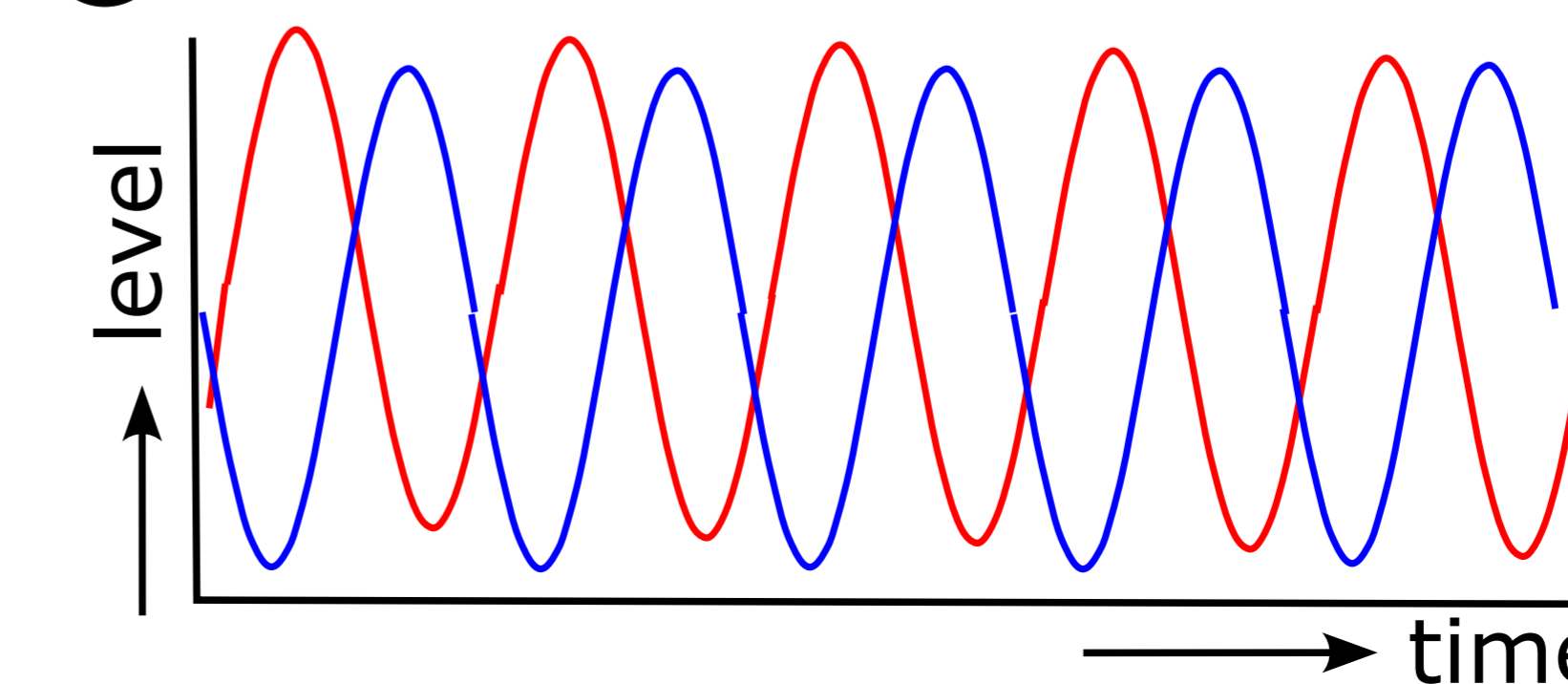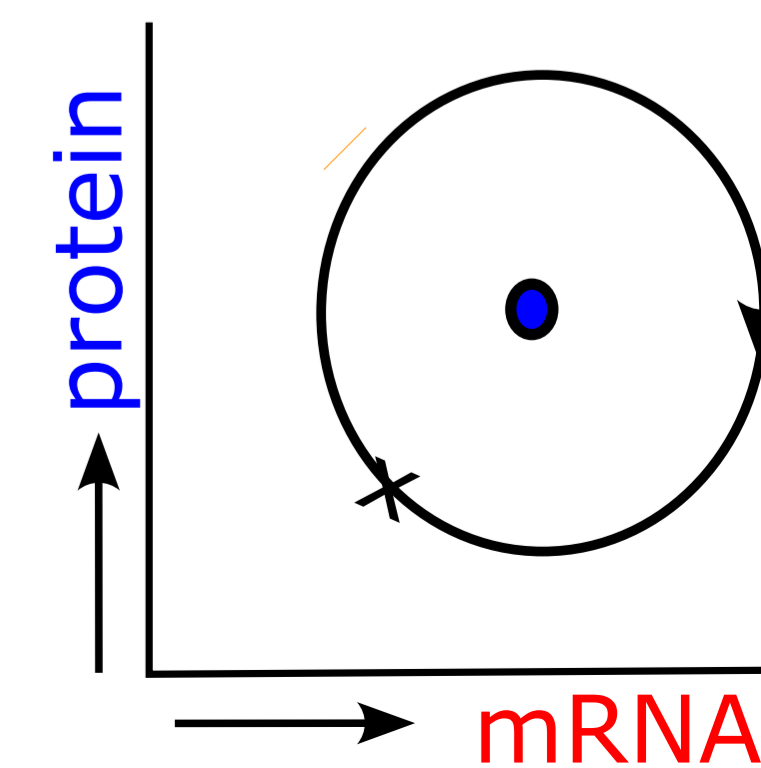

Supplement: koag213_Supplementary_Data [file koag213_supplementary_data.zip › SupplFig1_new.pdf]

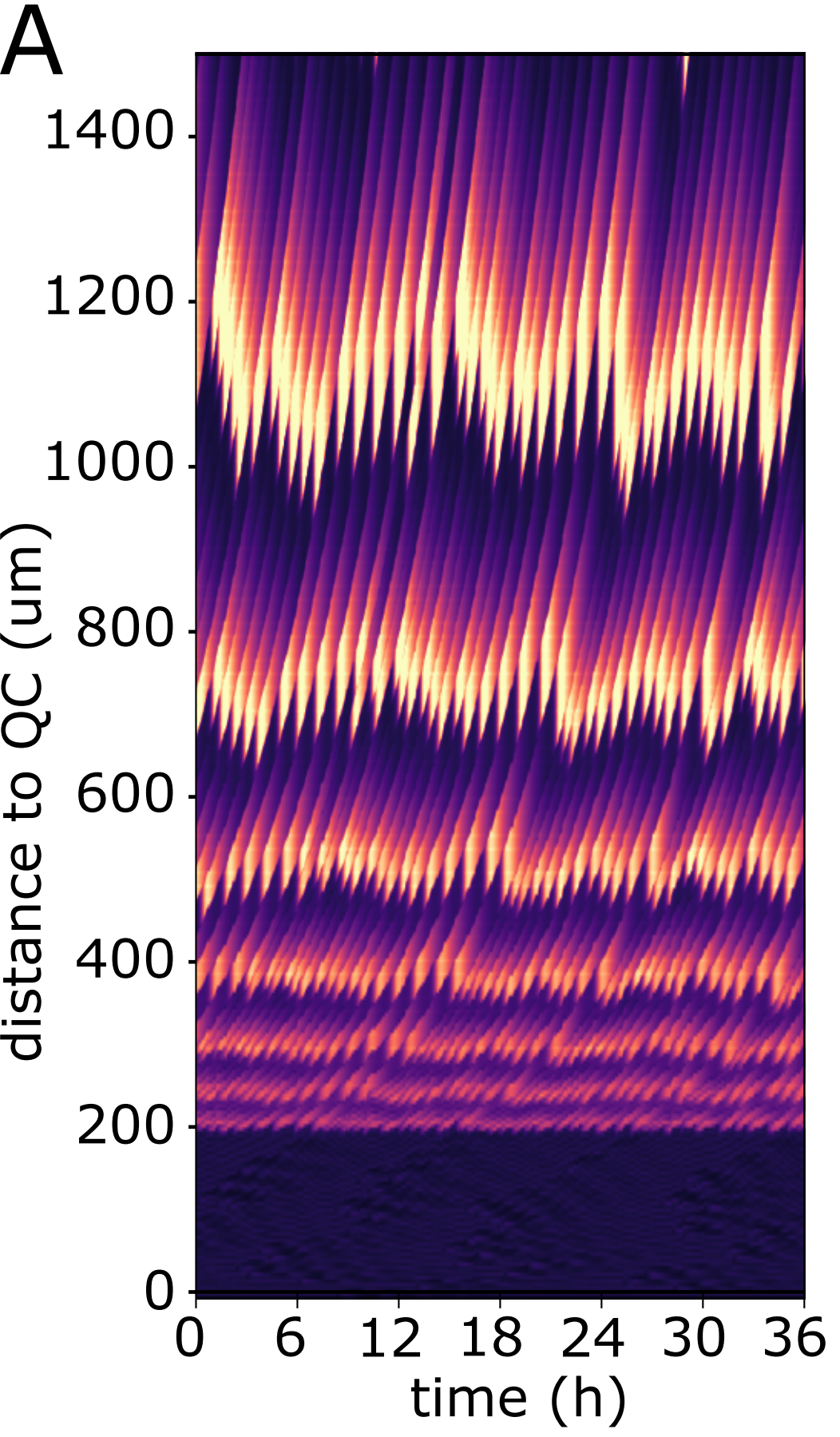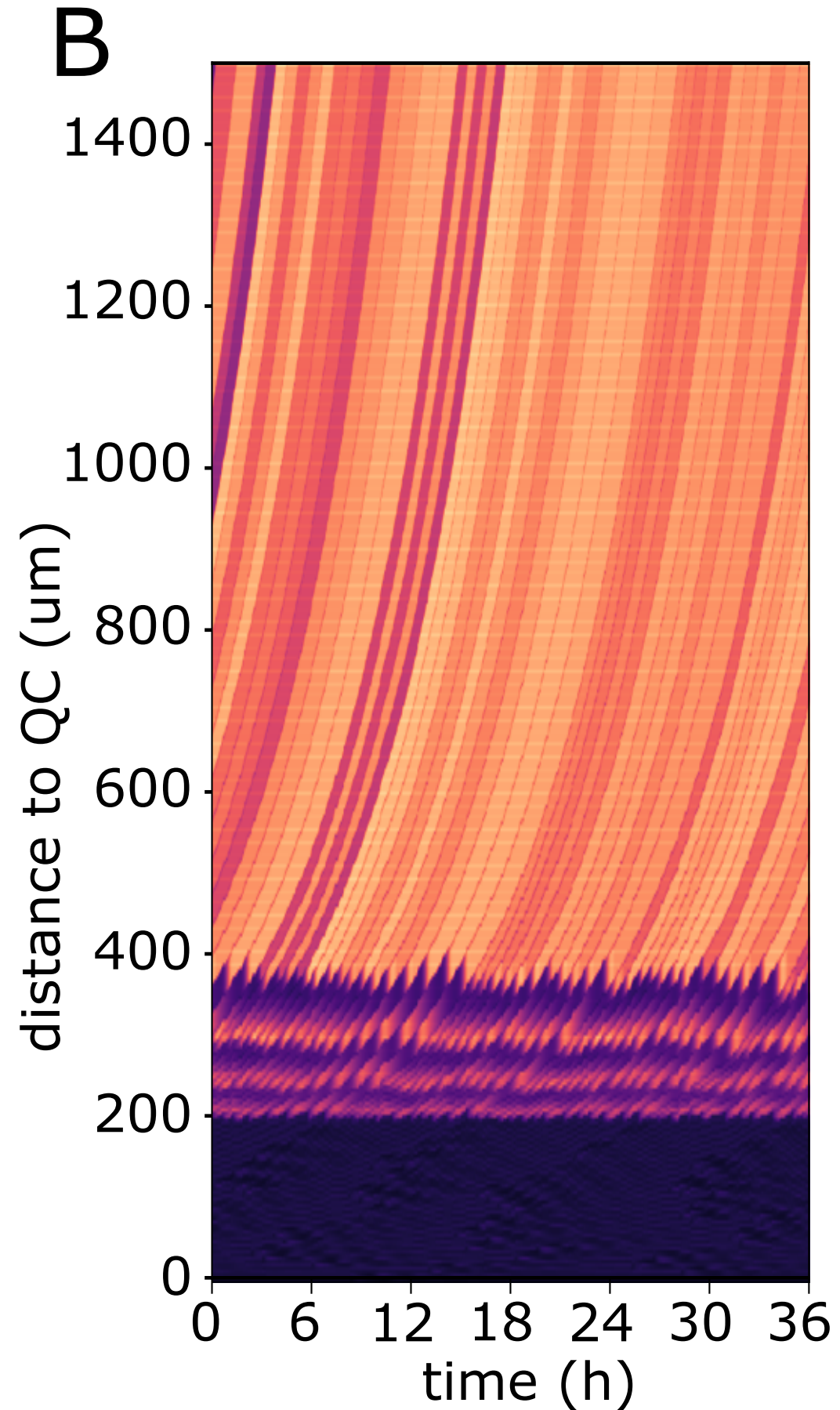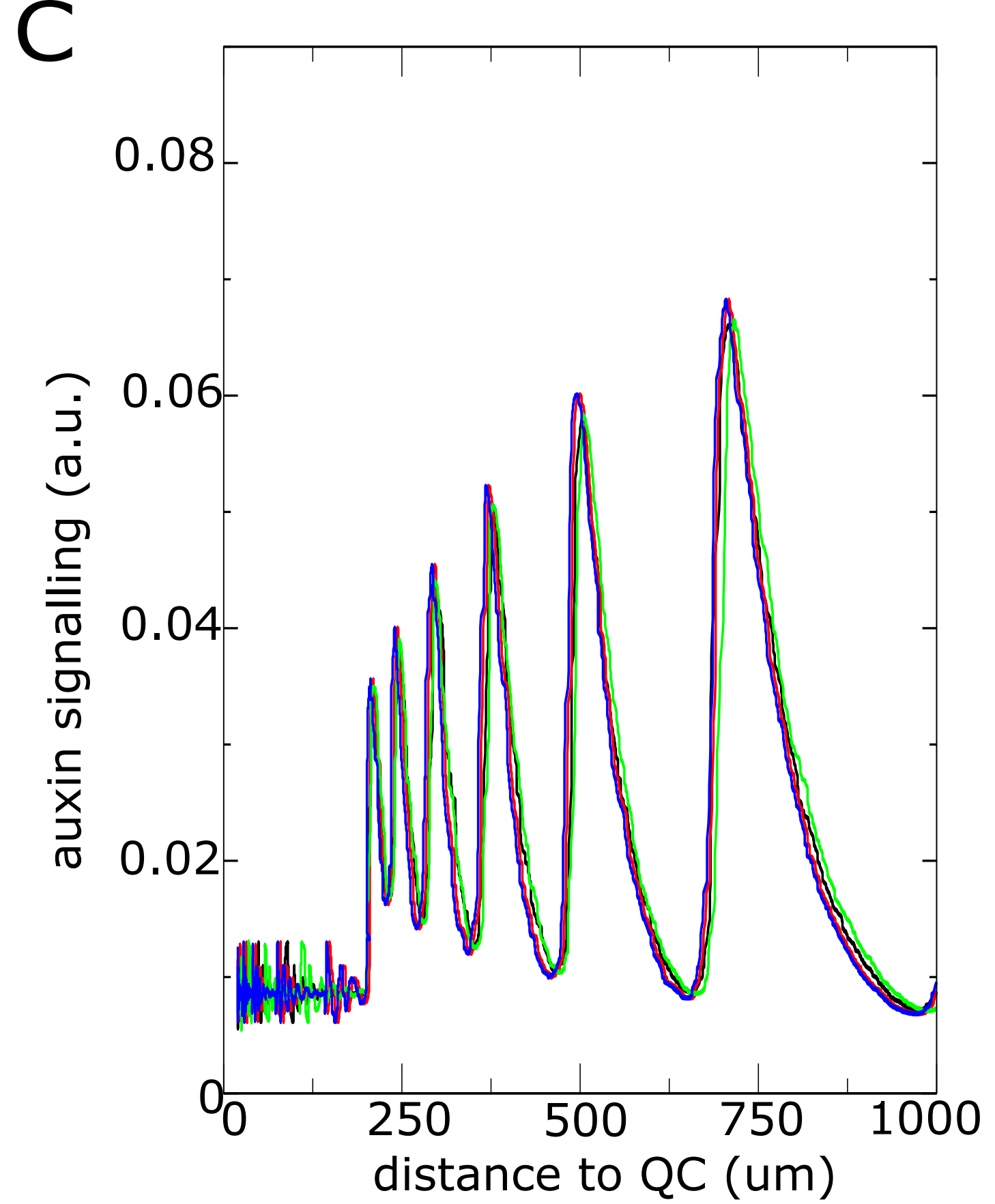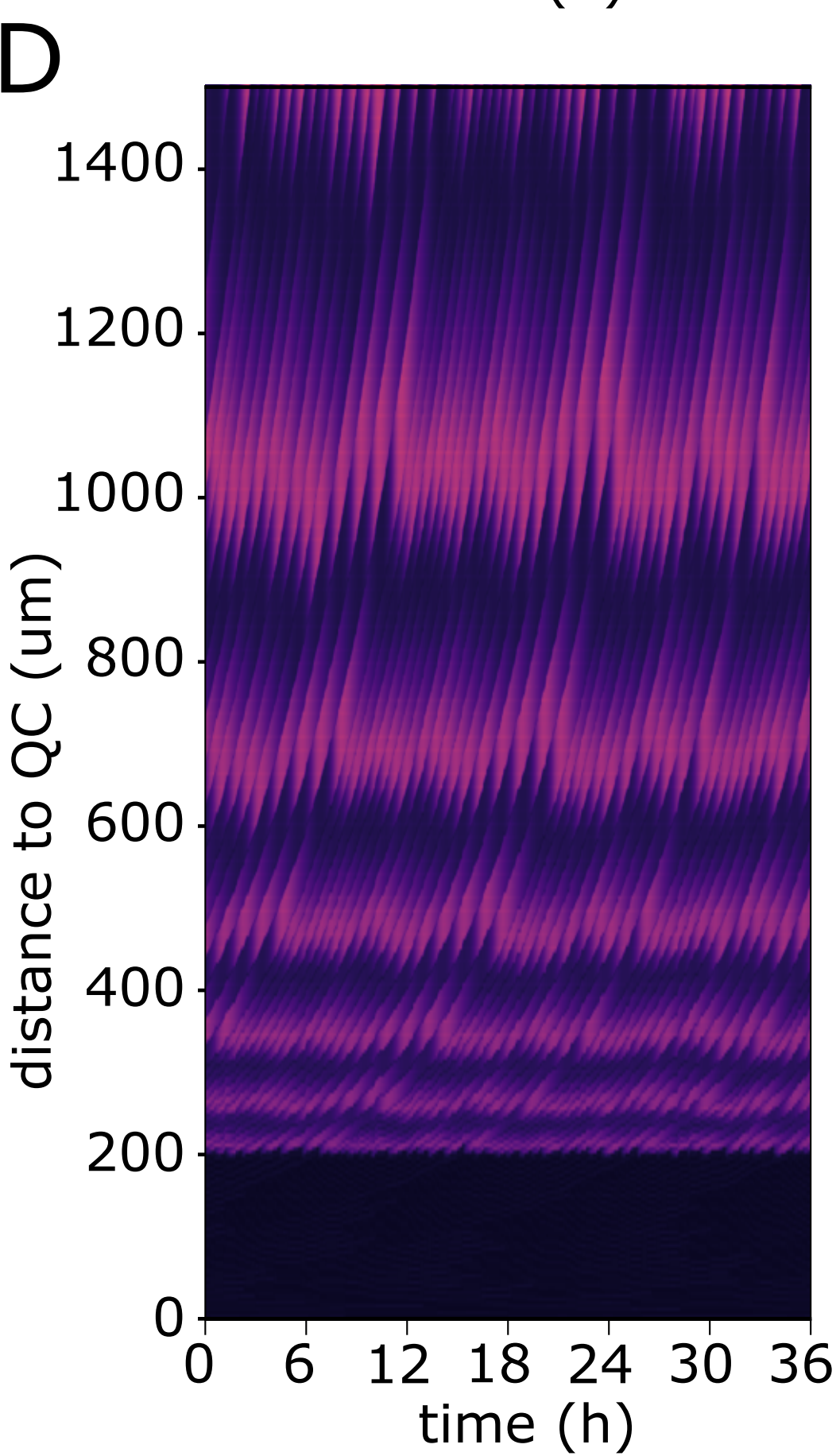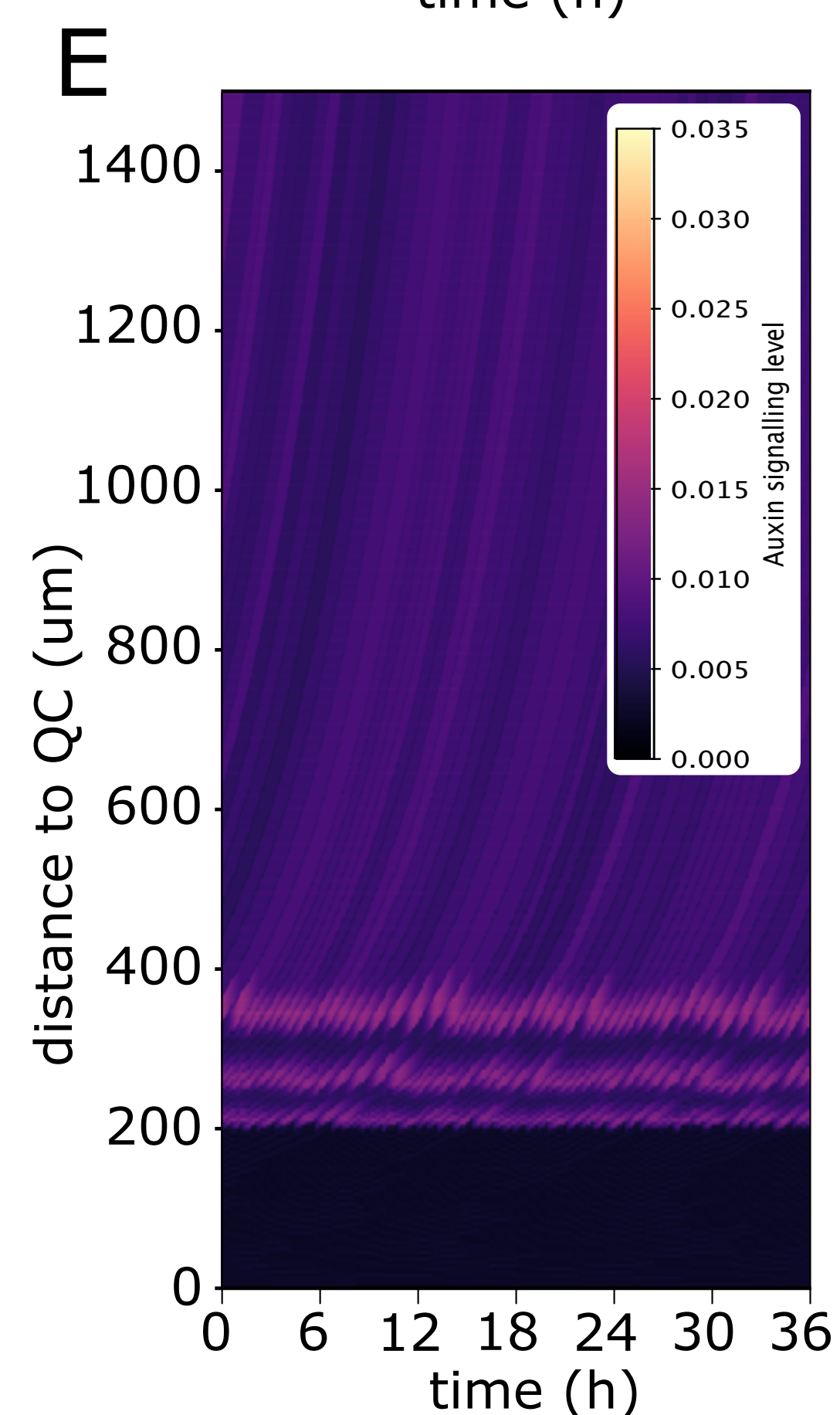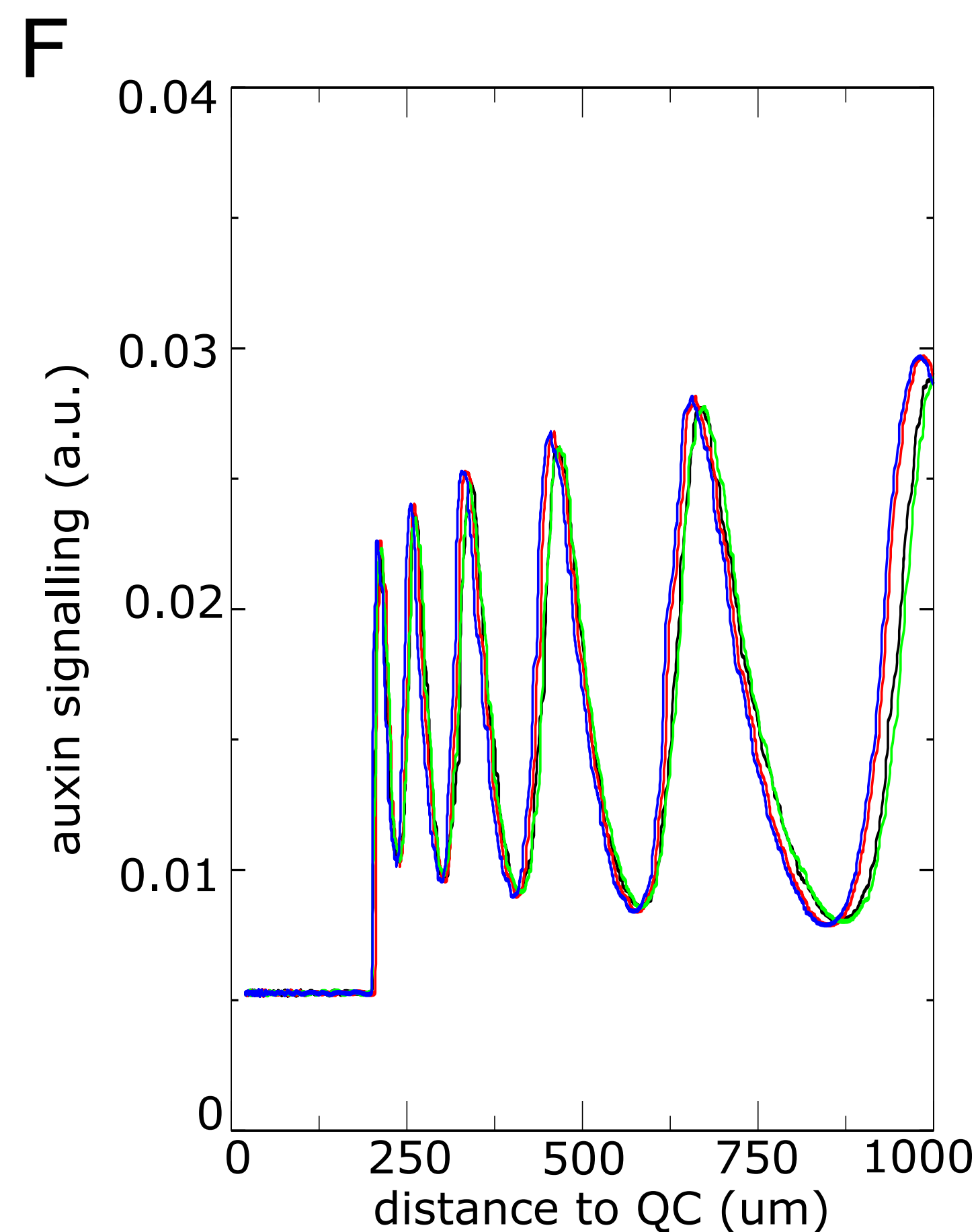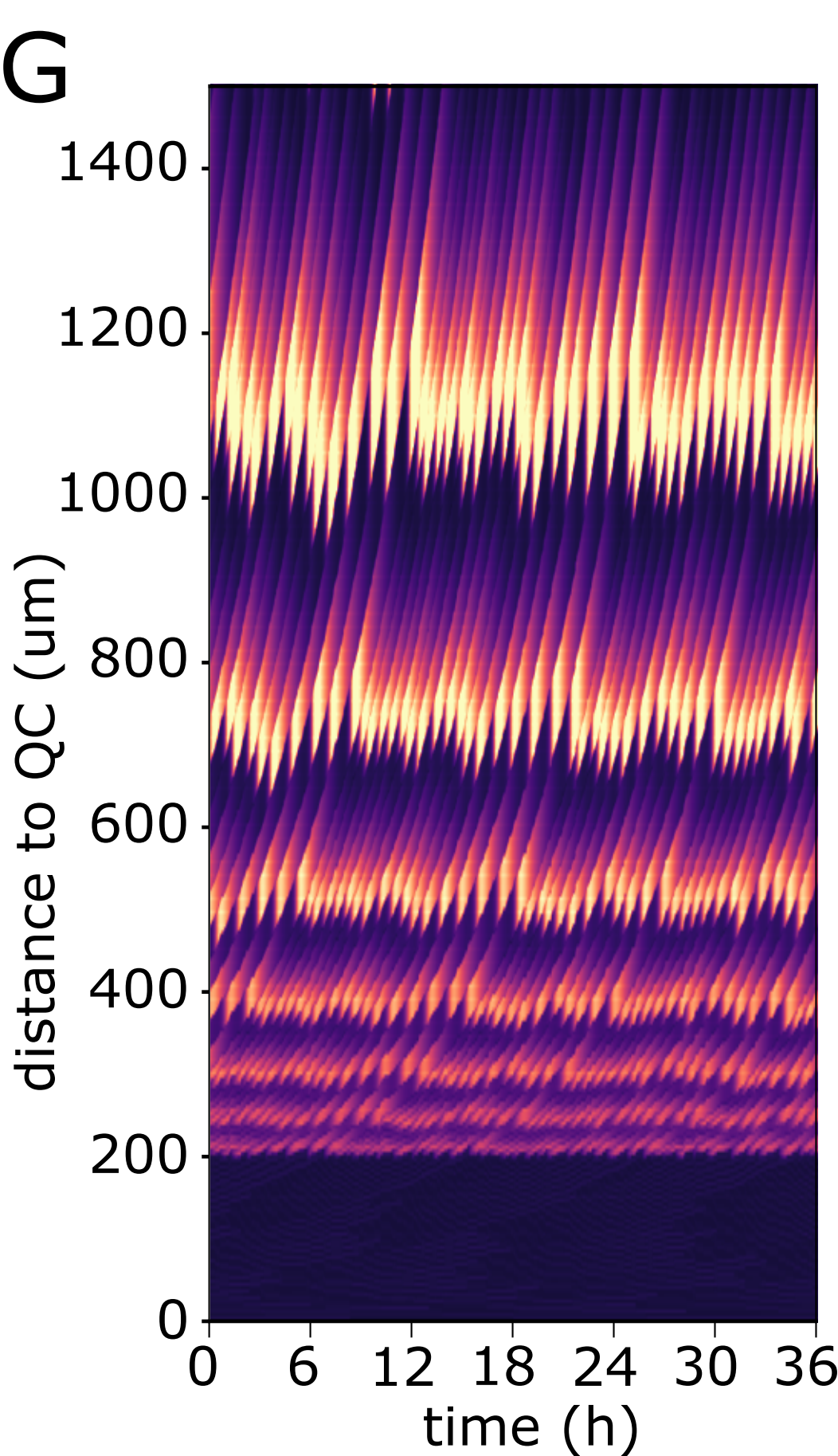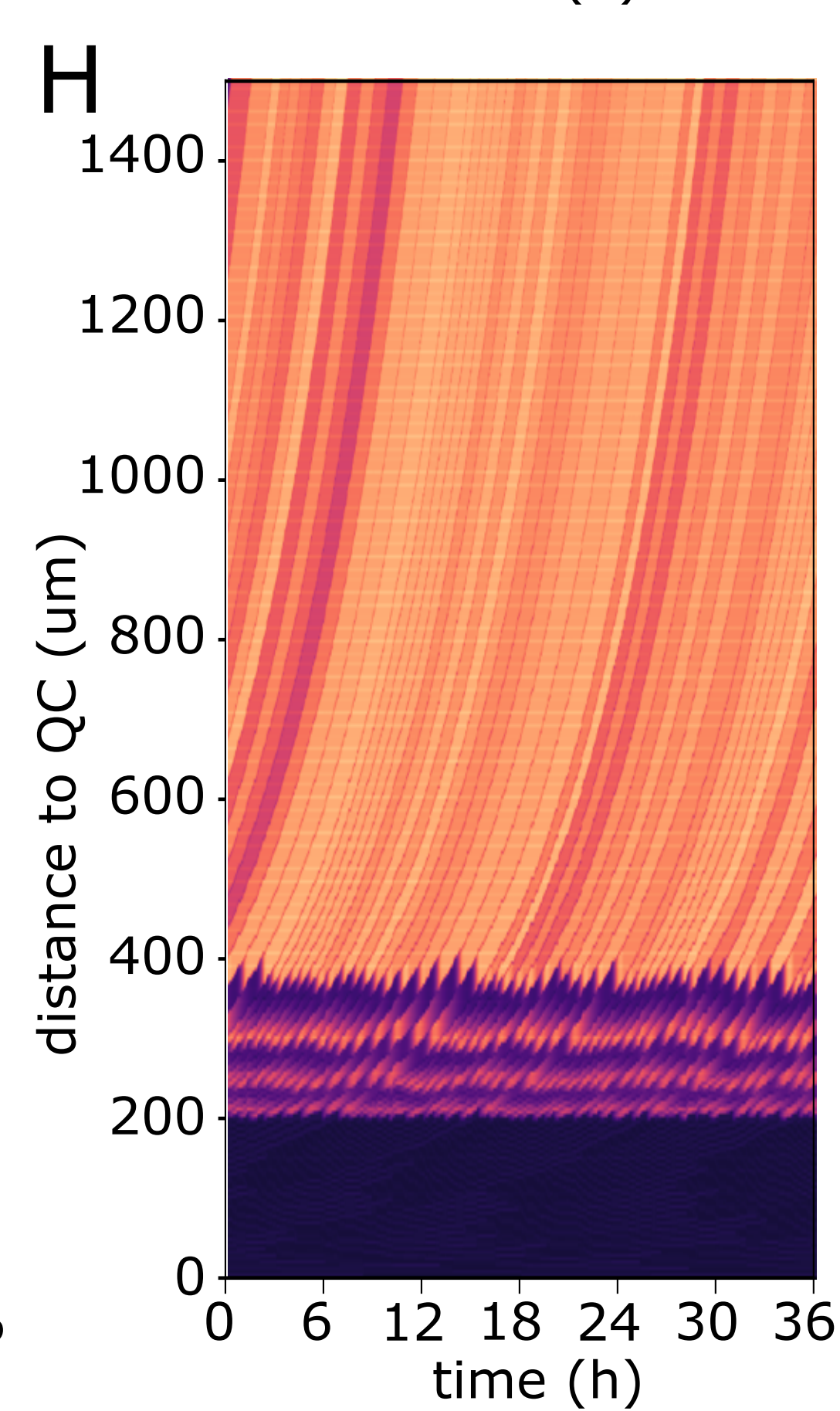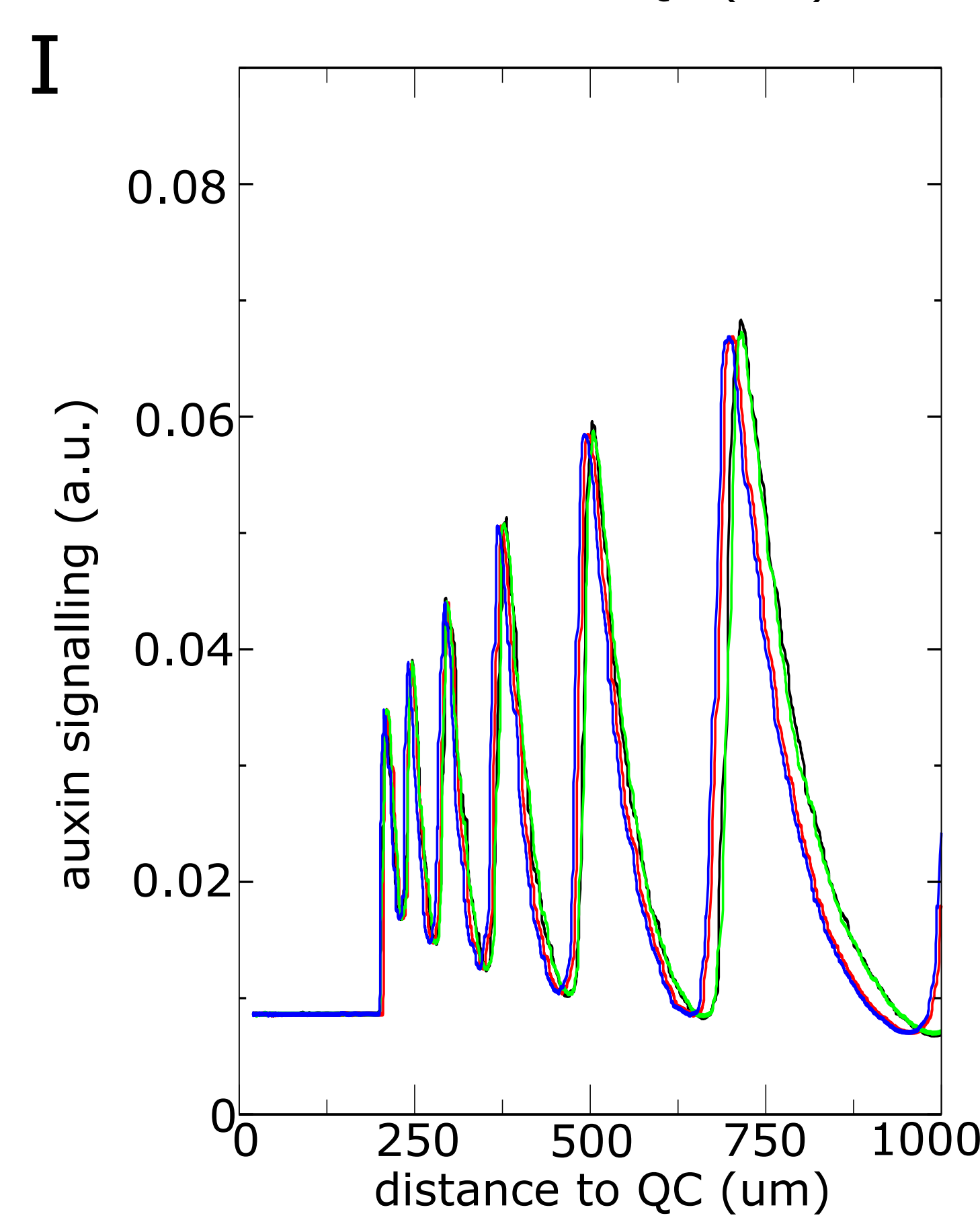

Supplement: koag213_Supplementary_Data [file koag213_supplementary_data.zip › SupplFig2_new.pdf]

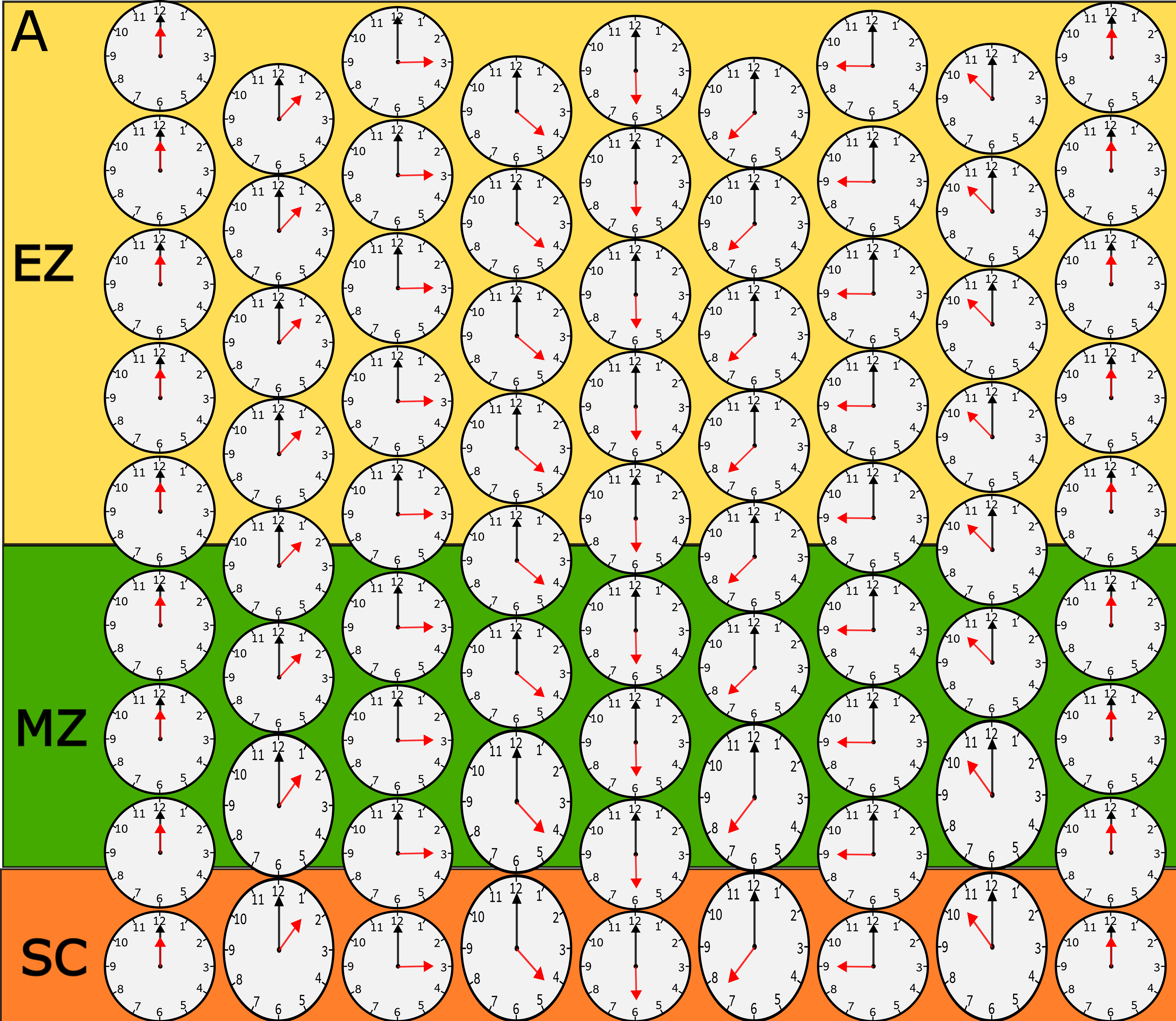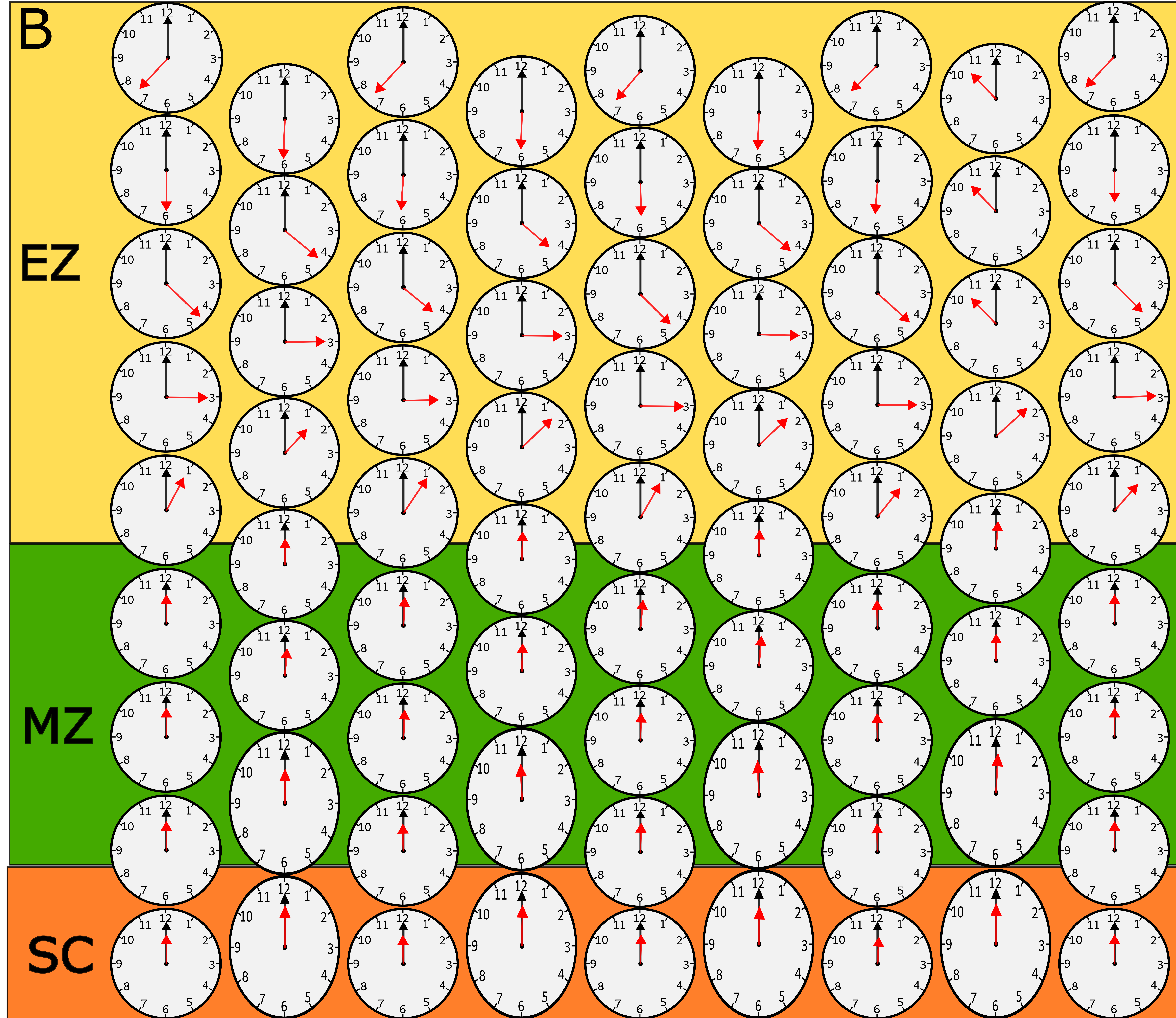

time

time

Supplement: koag213_Supplementary_Data [file koag213_supplementary_data.zip › SupplFig3_new.pdf]

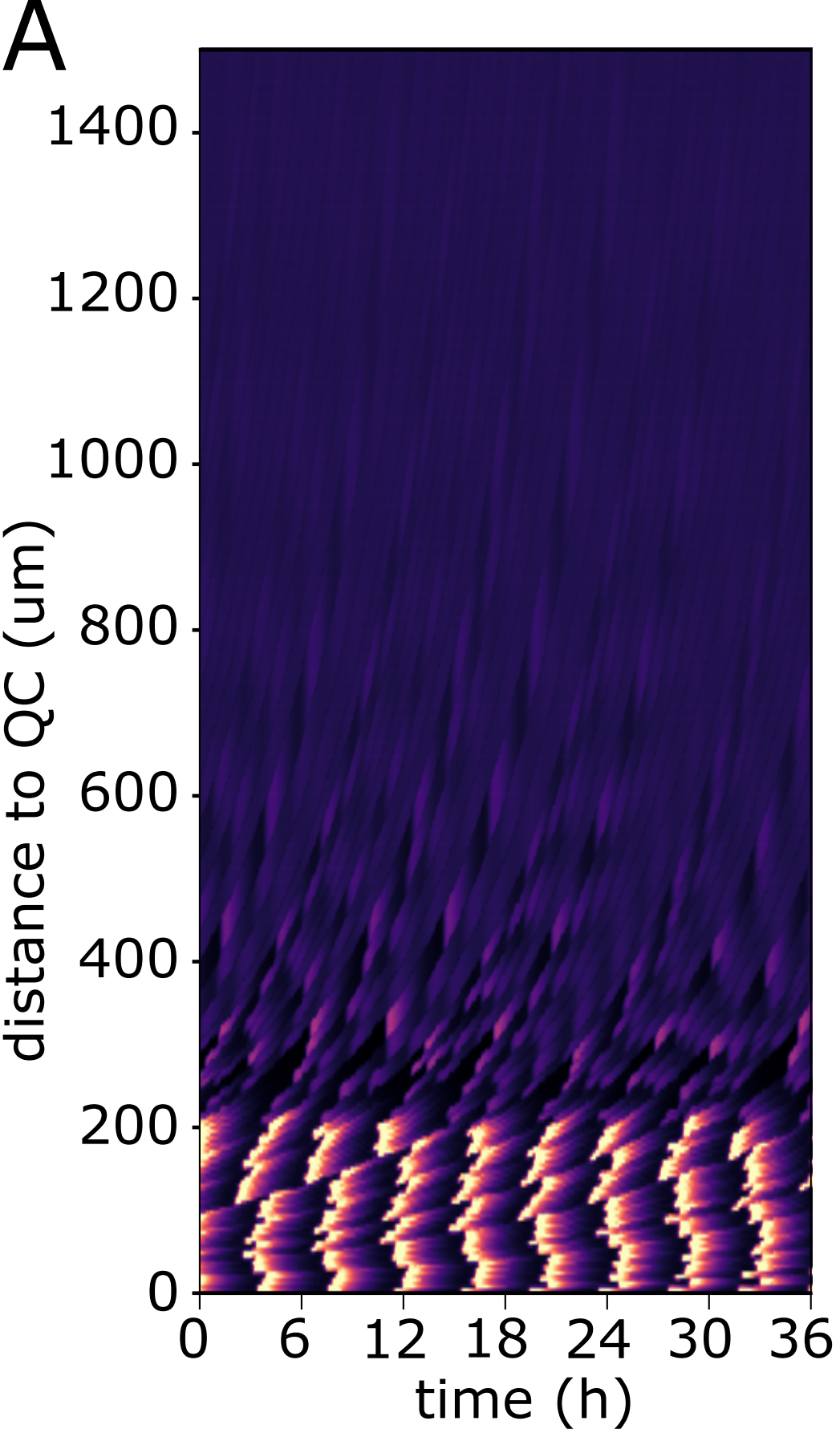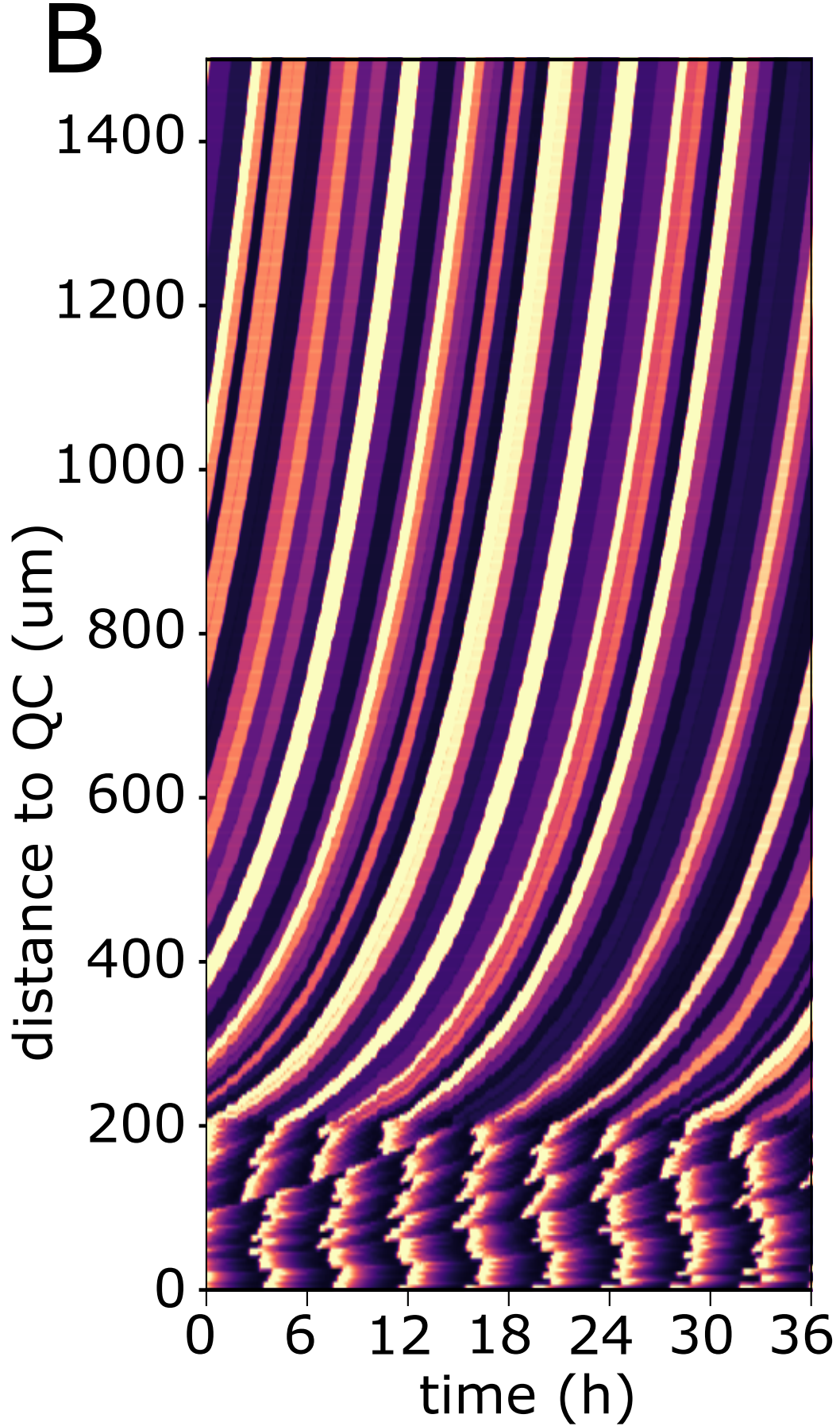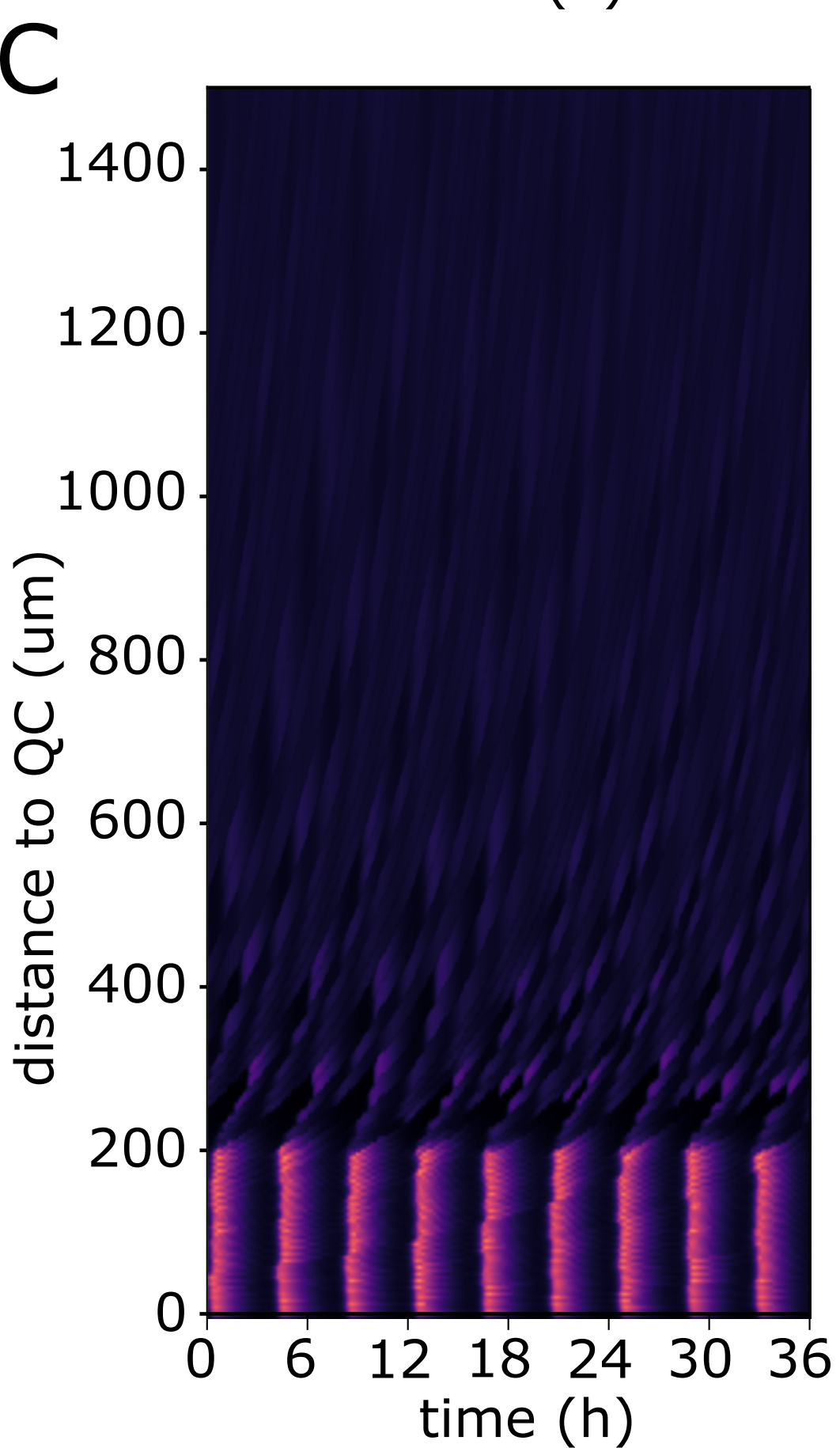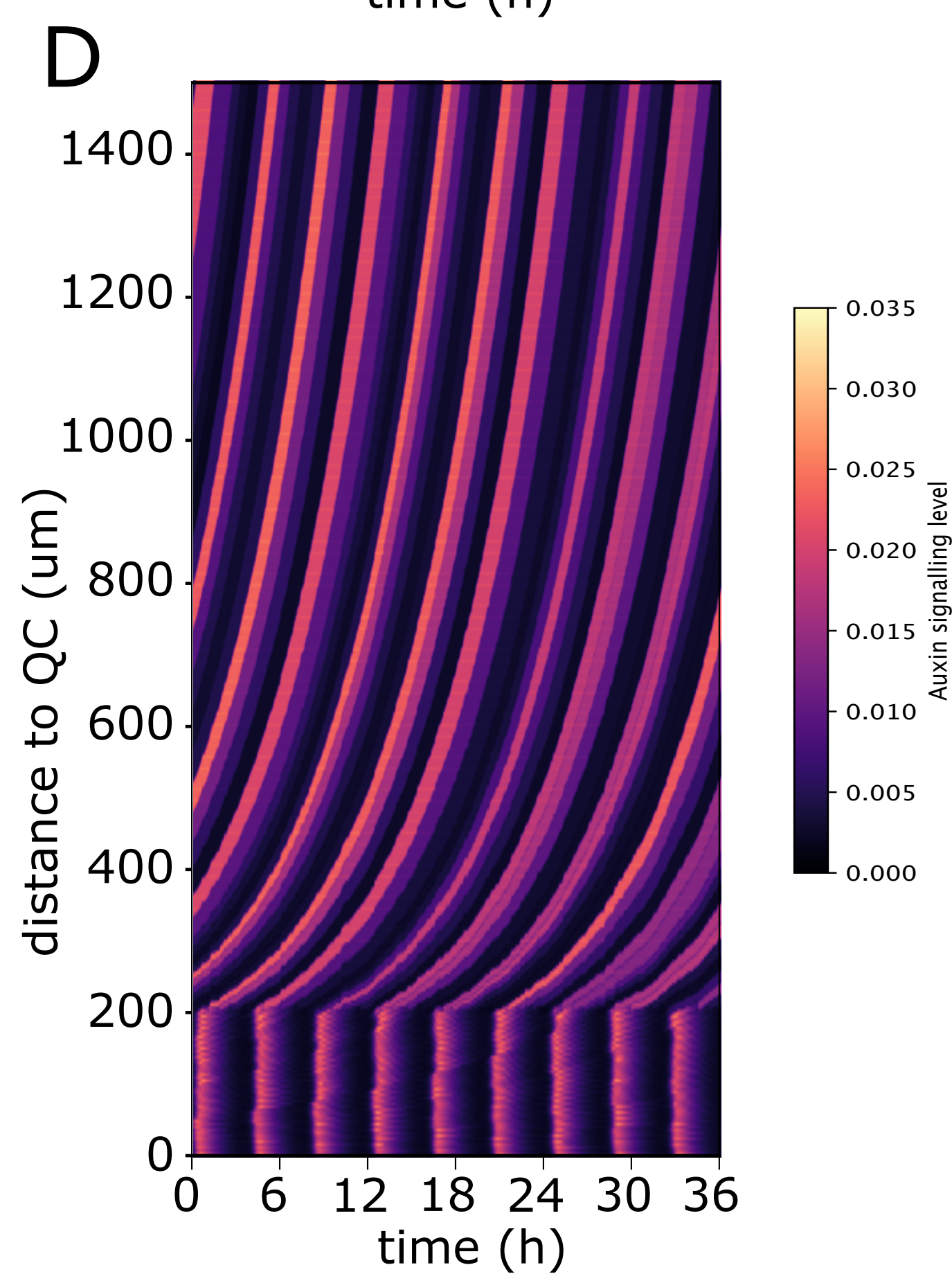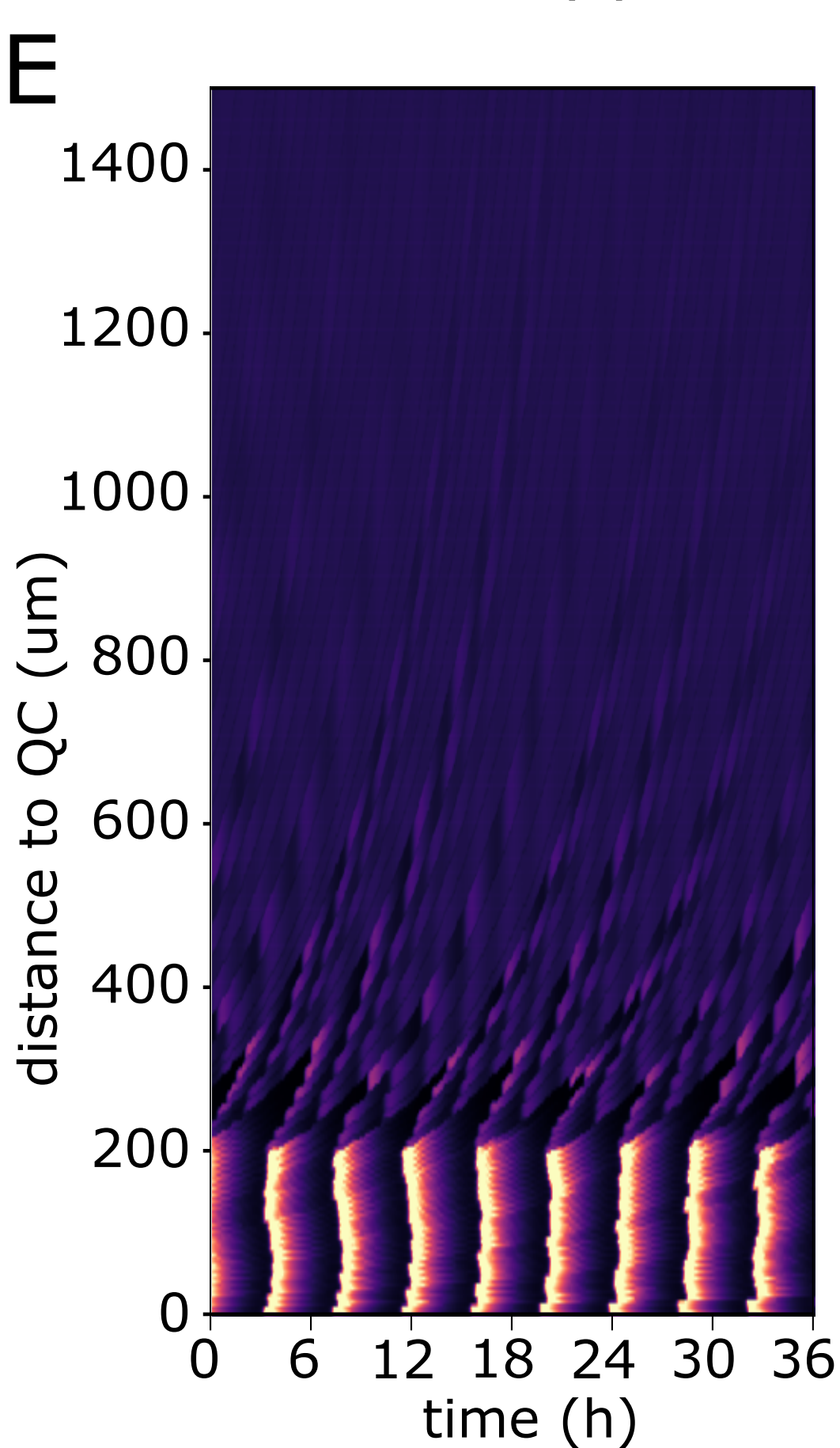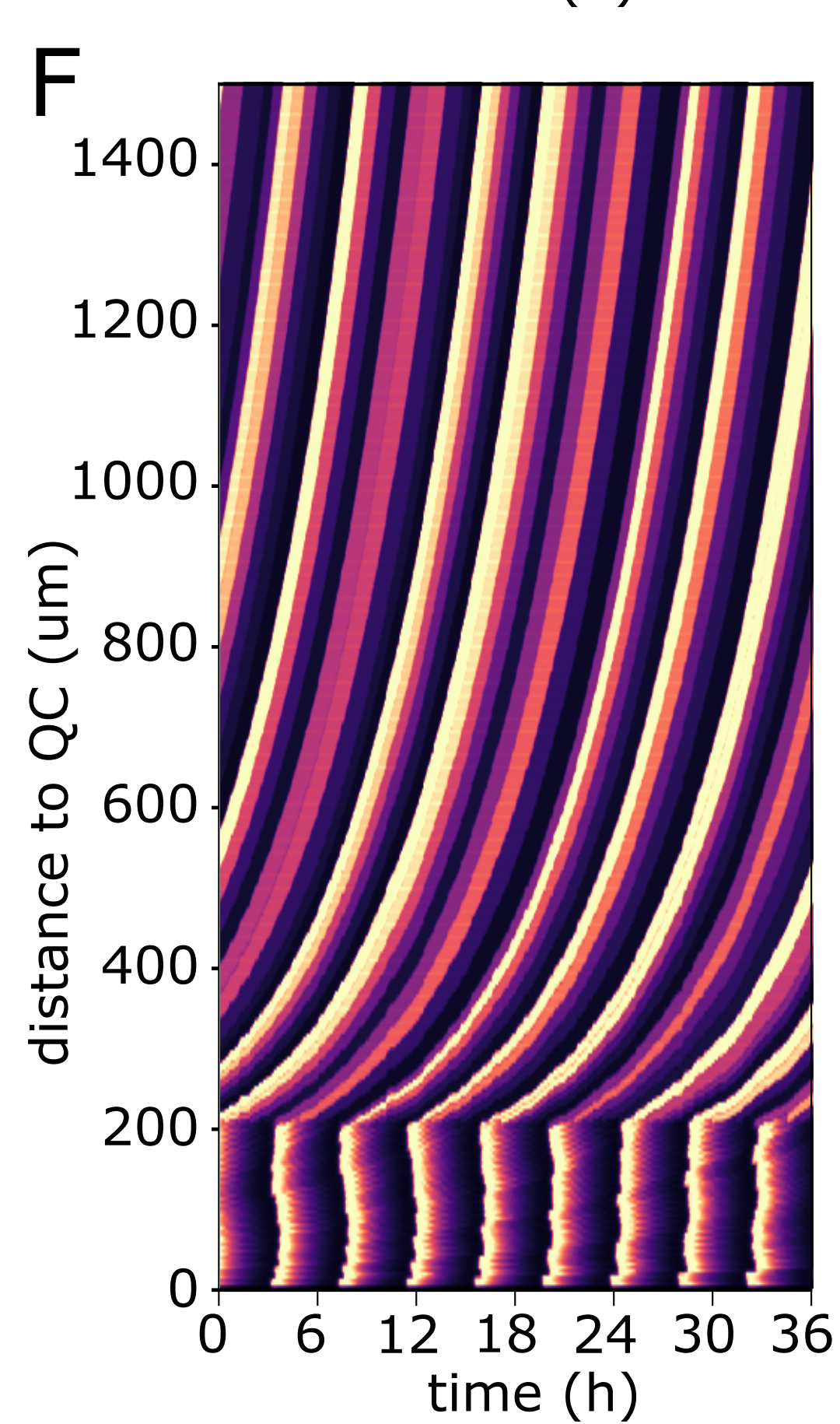

Supplement: koag213_Supplementary_Data [file koag213_supplementary_data.zip › SupplFig4_new.pdf]

**A**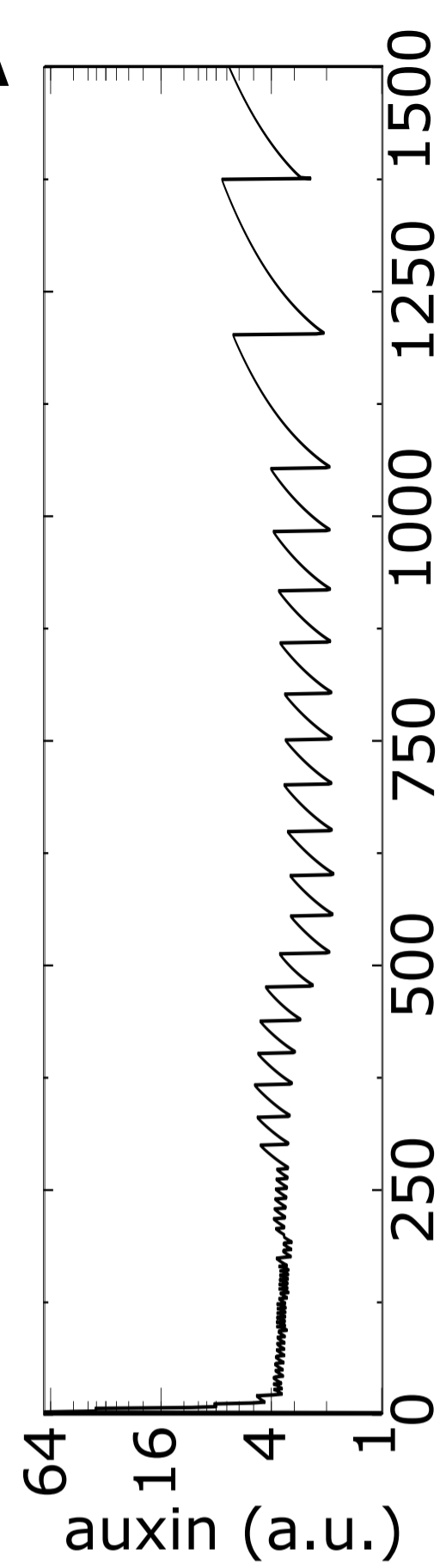**B**

distance to QC (μm)

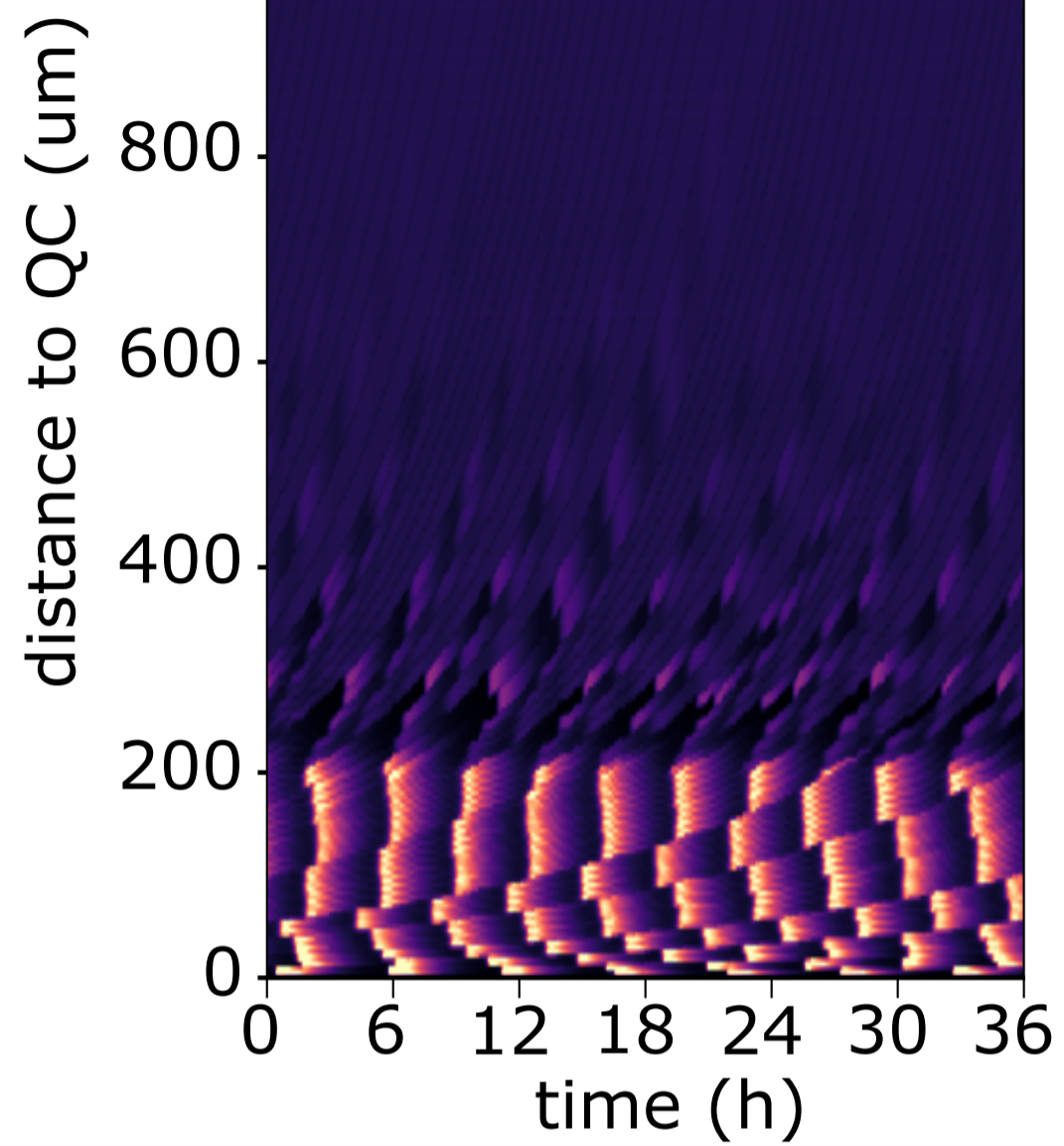**C**

distance to QC (μm)

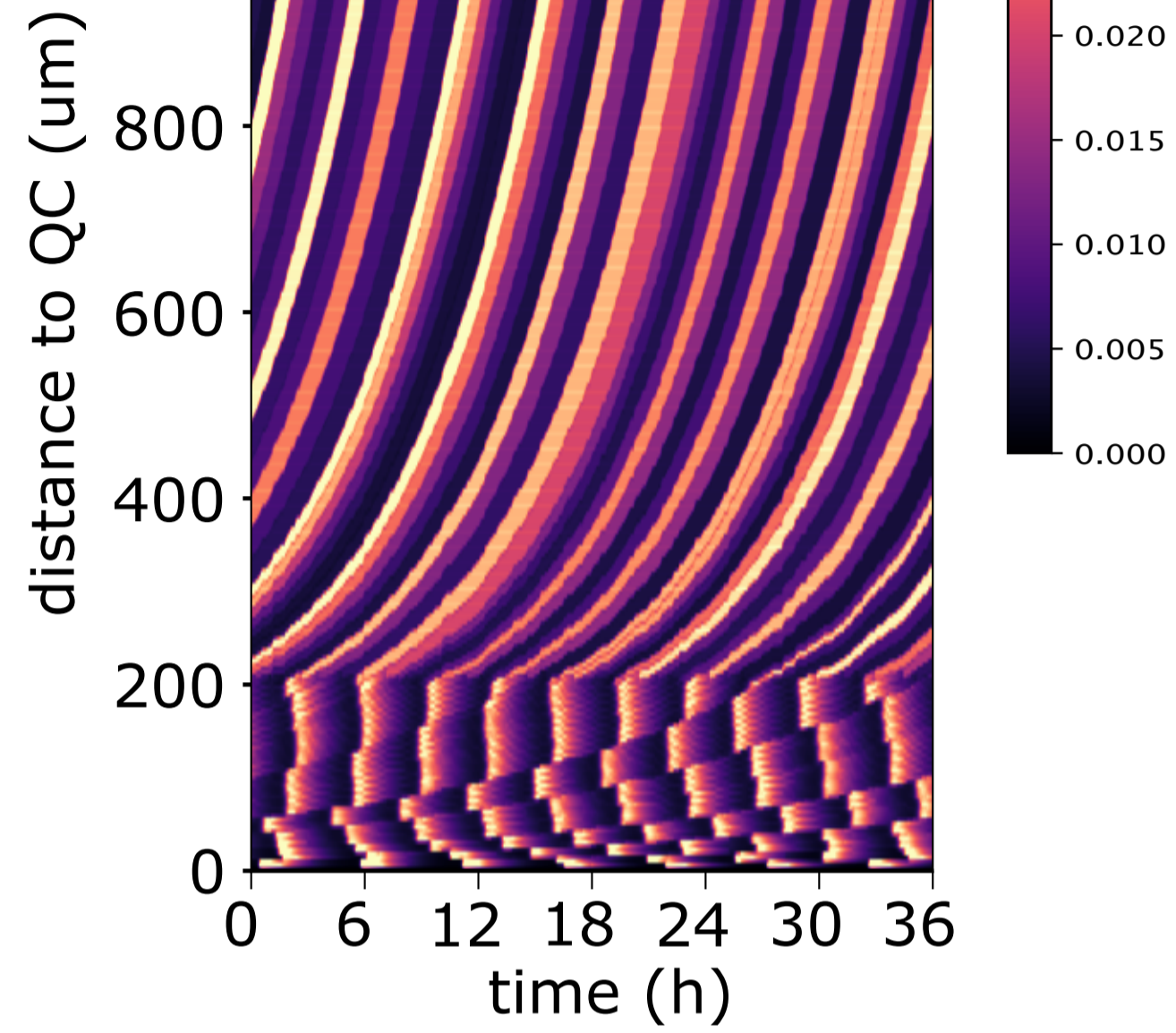**D**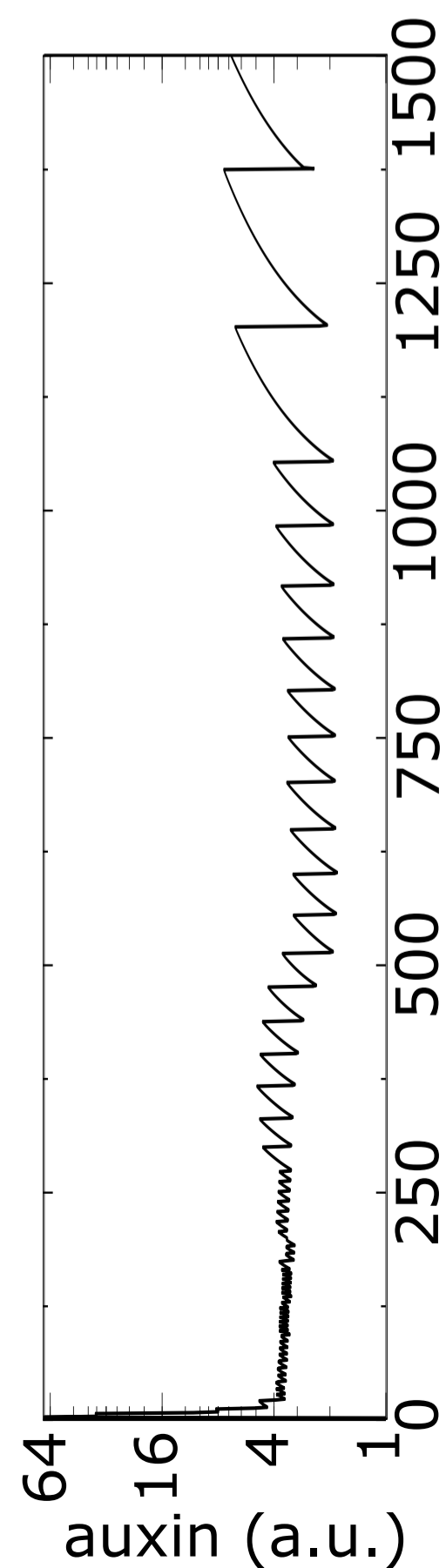**E**

distance to QC (μm)

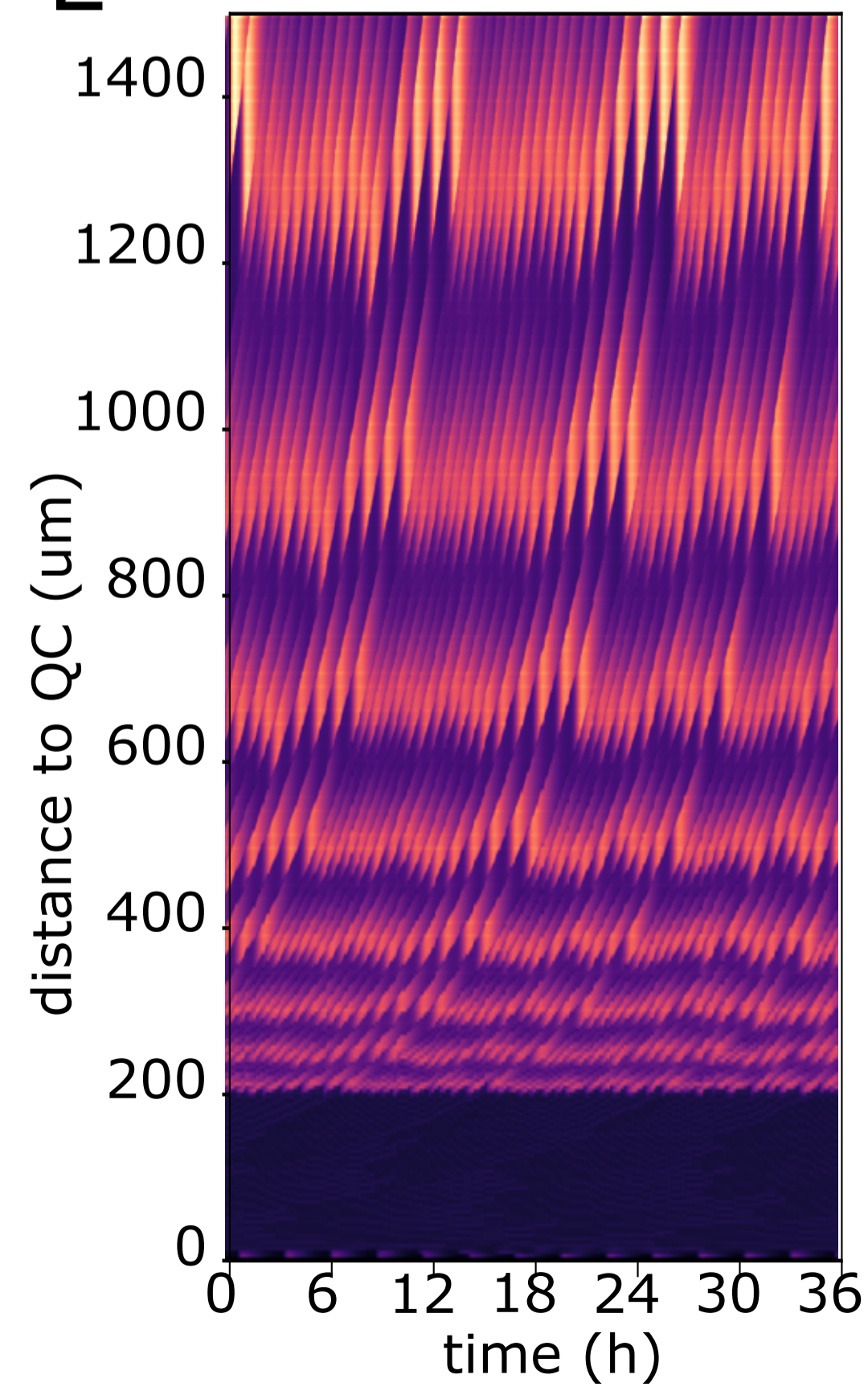**F**

distance to QC (μm)

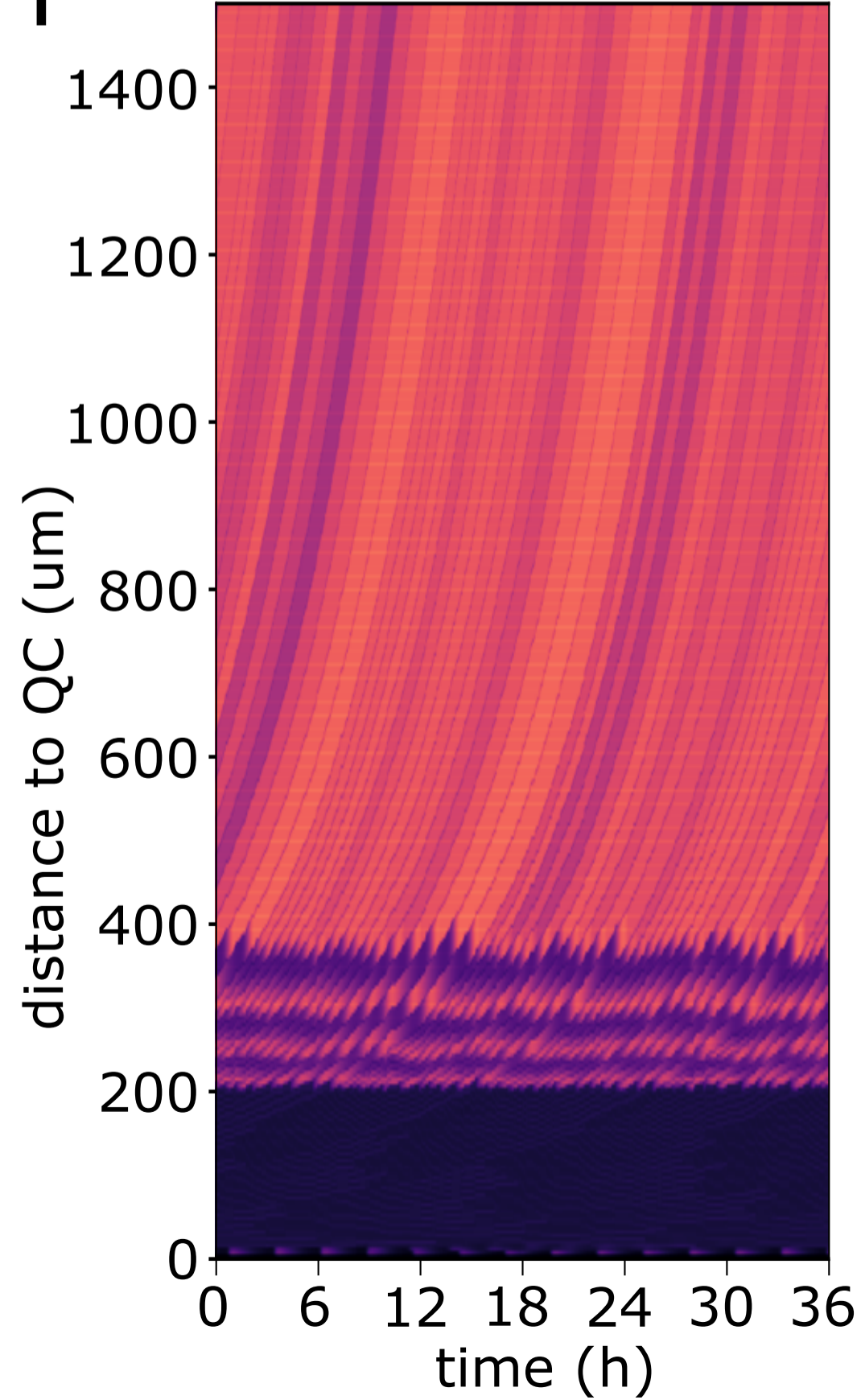

Supplement: koag213_Supplementary_Data [file koag213_supplementary_data.zip › SupplFig6_new.pdf]
